# The Influence of Ion Solvation and Association Interactions on Mean Ionic Activity Coefficients in Neutral Polymeric Membranes

**DOI:** 10.1021/acs.macromol.5c01588

**Published:** 2025-11-05

**Authors:** Sean M. Bannon, Rachel L. Fetter, Viatcheslav Freger, Geoffrey M. Geise

**Affiliations:** † Department of Chemical Engineering, 2358 University of Virginia, 385 McCormick Road, Charlottesville, Virginia 22903, United States; ‡ Wolfson Department of Chemical Engineering, 26747 Technion - Israel Institute of Technology, Haifa 32000, Israel

## Abstract

The influence of material properties on ion-hydrated polymer thermodynamic interactions is not fully understood. In this study, we probed how polymer properties (i.e., the network mesh size and dielectric constant) contribute to interactions between ions, water molecules, and the solvated polymer by synthesizing polymer networks with varied cross-link density and functionality (e.g., hydroxyl, ether, or nitrile). We characterized the hydration-dependent network mesh size, relative permittivity (i.e., dielectric constant), and the sodium chloride mean ionic activity coefficients in the polymers and related these properties to each other using a theoretical model that describes quantitatively the influence of ion solvation and ion pairing interactions on ionic activity coefficients in neutral polymers. These results and analysis help to explain the relationship between the network mesh size, polymer dielectric constant, and mean ionic activity coefficients in solvated polymers, which may be useful to guide molecular engineering strategies for polymer membrane materials.

## Graphical abstract

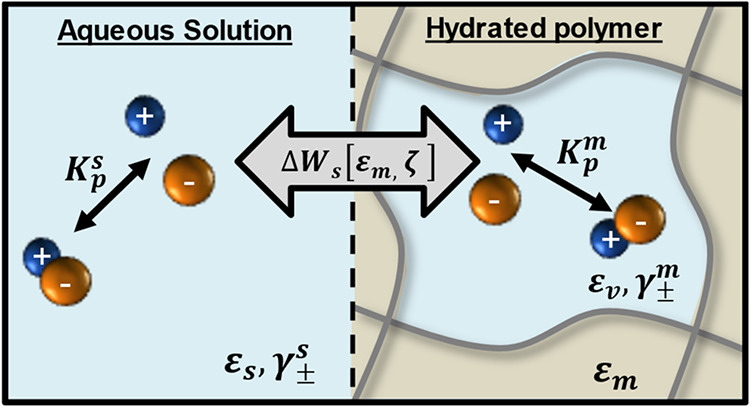

## 1 Introduction

Many separation (e.g., desalination)^1–3^ and biological (e.g., drug delivery)^4–6^ processes require the equilibration of a hydrated polymer with an aqueous electrolyte solution. In these processes, ions and water partition into the polymer from the electrolyte to minimize the Gibbs free energy between the solution and polymer, and under these circumstances, the equilibrium concentration of ions in the polymer is effectively governed by differences (between solution and polymer) in thermodynamic nonideality.^7–9^ As a result, developing a detailed understanding of how polymer properties contribute to ionic thermodynamic nonideality would be useful to better understand strategies to engineer a variety of biological devices and separation materials.

In electrolyte solutions, thermodynamic nonideality is quantified using mean ionic activity coefficients.^10^ Mean ionic activity coefficients describe how interactions between cations, anions, and their solvated environment contribute to the total free energy of the electrolyte solution, and are defined as^11^

1 $$\gamma_{\pm }^{j}=\exp\left[\frac{{\mathrm{G̅}}_{\pm }^{E,j}}{\mathrm{RT}}\right]$$

where *G̅*_±_^*E, j*^ is the mean ionic excess partial molar Gibbs free energy of the electrolyte in phase *j* (defined as *G̅*_±_^*E, j*^ = ∑_*i*_ *v*_*i*_ *G̅*_*i*_^*E, j*^/∑_*i*_ *v*_*i*_), and *RT* is the molar thermal energy (i.e., the product of the gas constant and absolute temperature). Within the context of eq 1, when the mean ionic activity coefficients of an electrolyte are unity, the mean ionic excess partial molar Gibbs free energy is zero, which corresponds to ideal interactions. Alternatively, when the mean ionic activity coefficients are greater than unity, the excess partial molar Gibbs free energy is greater than zero (i.e., interactions are thermodynamically nonfavorable and increase the total free energy of the system relative to the ideal state), and when the mean ionic activity coefficients are less than unity, the excess partial molar Gibbs free energy is less than zero (i.e., interactions are thermodynamically favorable and reduce the total free energy of the system relative to the ideal state). Because of this relationship between ion activity coefficients and the Gibbs free energy of aqueous solutions, the concentration of ions in hydrated polymers relative to that in the external solution is proportional to the ratio of the activity coefficients in solution and polymer.^7–9,12,13^

Often, these activity coefficients are significantly influenced by aspects of solution chemistry such as the dielectric constant, solution concentration, and ionic properties (e.g., charge and valence).^14–19^ One strategy to understand how solution chemistry contributes to mean ionic activity coefficients is to mathematically model them, using the tools of thermodynamics, by considering interactions between ions and solvent molecules. Historically, continuum-level thermodynamic theories such as the Born model,^20^ the Debye–Hückel limiting-law,^19^ and Bjerrum’s ion association theory,^21^ have been used to describe the nature and extent of specific electrostatic interactions in aqueous electrolyte solutions, such as ion solvation interactions (Figure 1A), and Coulombic ion–ion interactions (Figure 1B,C).^11^ These continuum-level theories have been useful as tools that describe the molecular underpinnings of how solution properties contribute to molecular interactions in dilute electrolytes, and contemporary models that describe quantitatively the mean ionic activity coefficients of electrolyte solutions over wide ranges of concentrations (e.g., the Pitzer model, which requires the use of electrolyte-specific tabulated parameters) often empirically describe how combinations of short- and long-range interactions contribute to mean ionic activity coefficients in solutions.^22^

**Figure 1:**
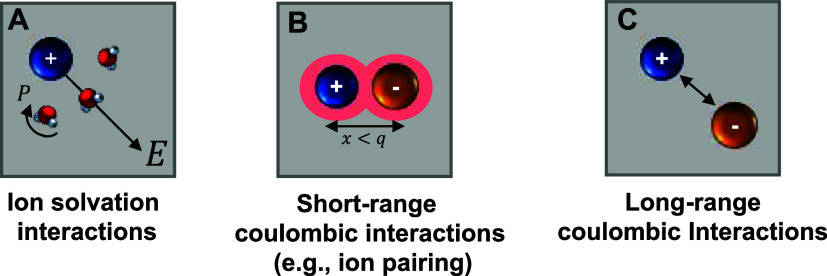
Depictions of types of interactions that can be important in electrolytes: (A) solvation interactions between ions (which create an electric field *E*) and solvent (which are displaced by a polarization, *P*), (B) short-range Coulombic interactions such as ion–ion association (i.e., pairing) of two ions that are separated by a distance *x* that is less than the cutoff distance separating paired and free states, *q*, and (C) long-range Coulombic interactions that describe multi-ion interactions using mean-field approximations.

Because there are few water molecules per unit volume in a hydrated polymer relative to an aqueous electrolyte, many hydrated polymers containing dissociated ions can be approximated, in the simplest case, as concentrated electrolyte solutions (often, for polymers containing charged/ionic chemical functionality, which are commonly used as materials to study ionic interactions in hydrated polymers,^7,9,12,23,24^ the concentration of dissociated charges in the polymer is on the order of magnitude of 10° to 10^1^ [mol/L (water sorbed)]).^7,25,26^ Generally, the predictions of the classic models, discussed previously, exhibit significant discrepancies from experimentally determined ion activity coefficient data in concentrated electrolytes (i.e., >0.1 mol/L) because, under concentrated conditions, the magnitude and extent of electrostatic interactions is significantly more complex than in dilute systems.^11^ These complexities obscure understanding of how polymer chemistry contributes to ionic activity coefficients, and fundamental studies investigating the molecular underpinnings that relate intrinsic material properties to ionic interactions in hydrated polymers would be useful to address this knowledge gap.

Neutral polymers serve as model materials to understand the fundamental influence of material properties on ionic interactions in hydrated polymers, because relative to charge/ion-functionalized polymers, the concentration of ions dissociated in the polymer-sorbed solution is small (e.g., 10^–2^ to 10° [mol/L (water sorbed)]).^27^ In hydrated neutral polymers, it is commonly observed that mean ionic activity coefficients are greater than unity (i.e., are thermodynamically nonfavorable) and decrease in magnitude with increasing water volume fraction (Figure 2).^28^ Relative to aqueous sodium chloride solutions, where the activity coefficients are generally less than unity (at modest concentrations),^17^ these observations suggest that thermodynamic ionic interactions in hydrated polymers are generally more nonfavorable than those in aqueous electrolytes.

**Figure 2:**
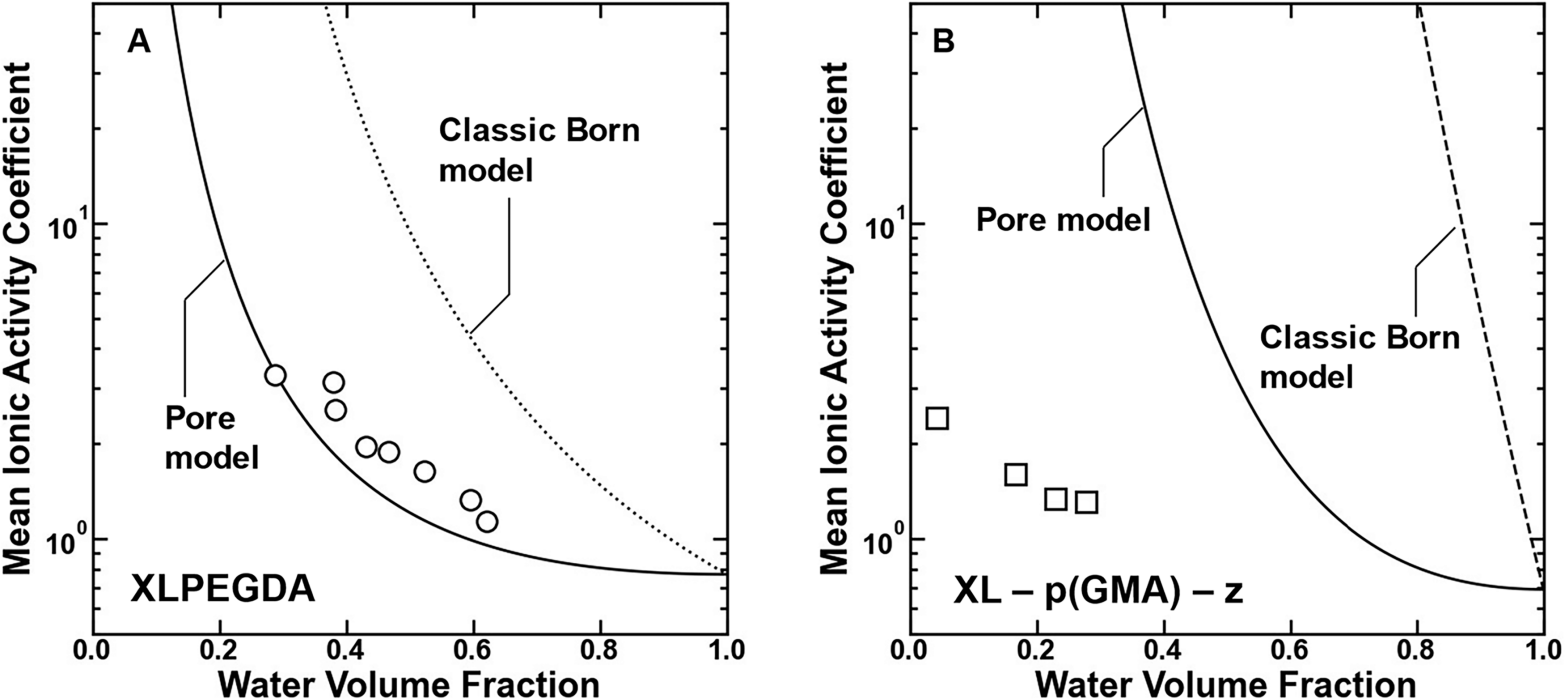
Mean ionic activity coefficients of sodium chloride in (A) XLPEGDA^8^ and (B) XL – p­(GMA – z)^28^ that were equilibrated with 0.1 M NaCl and 0.5 M NaCl, respectively, plotted as a function of the polymer water volume fraction. Freger’s pore model (solid line) and the classic Born model (dashed line) are applied to describe the influence of ion solvation interactions on mean ionic activity coefficientsSupporting Information

These observations can be explained, to a first approximation, by a physical picture where ion solvation interactions (i.e., interactions between ions and their induced polarization charges, or so-called self-interactions (Figure 1A)) are nonfavorable in hydrated polymers relative to aqueous electrolytes. Ion solvation interactions in electrolyte solutions are primarily governed by the solvent (i.e., water or the hydrated polymer matrix) dielectric constant, because high dielectric constant solvents have many polarization charges that can contribute to stabilizing ion solvation (i.e., dissociation).^29^ The dielectric constant of a hydrated polymer is generally less than that in a bulk solution, and increases with increasing water volume fraction,^28,30–32^ and within this physical picture, ion solvation is expected to be increasingly favorable in high water volume fraction polymers where the dielectric constant is larger.

The influence of the ion solvation interactions on mean ionic activity coefficients in electrolyte solutions can be described using the Born model,^20^ which suggests that mean ionic activity coefficients of dissociated ions increase as the dielectric constant of the solvent is reduced.^7,8^ Classically, the Born model is applied to electrolyte solutions by assuming that the solvent can be described as a dielectric continuum, and determining the energy associated with solvent polarization in the presence of an ion. Using an appropriate relationship to relate the dielectric constant to the polymer water volume fraction (e.g., the Maxwell-Garnett model (Section S1)), the Born model describes qualitatively the expected scaling relationship between the water content and mean ionic activity coefficients (Figure 2). However, the Born model generally overestimates the experimentally observed mean ionic activity coefficients of hydrated polymers by several orders of magnitude (Figure 2). Different measures of ionic radii (e.g., cavity, bare, or solvated ionic radius) have complicated the application of the classic Born model to describe ion solvation interactions as well,^33–35^ but it is generally hypothesized that the classic Born model may be too simple to fully describe electrostatic interactions in hydrated polymers.^29^

The discrepancies between the Born model predictions and experimental activity coefficient data are, to a first-order approximation, a result of the dielectric continuum approximation used to derive the classic Born model.^8,36^ At sufficiently small length scales, polymers are phase-segregated materials that contain distinct hydrated void spaces dispersed throughout the polymer matrix, and therefore, hydrated polymers may be more accurately described as heterogeneous dielectric materials. Recently, Freger proposed the so-called pore model that can be used to describe the influence of this nanophase segregation on the thermodynamic environment of a hydrated polymer.^36^ In Freger’s pore model, the mean ionic activity coefficients of ions in the polymer can be estimated by placing ions at the center of the hydrated void and calculating the energy associated with the polarization of the surrounding heterogeneous dielectric.^36^ Effectively, this so-called pore model can be used to derive an expression for the mean ionic activity coefficient as a function of the polymer dielectric constant and the characteristic size of the hydrated void.^8^

Recently, we demonstrated that the pore model description of ion solvation interactions can be used to describe accurately the mean ionic activity coefficients of cross-linked poly­(ethylene glycol diacrylate)-based hydrogel materials (referred to as XLPEGDA) by using the characteristic network mesh size, which is also hydration-dependent, as an estimate for the hydrated void space (Figure 2A).^8^ Despite this success, we have observed significant discrepancies between pore model descriptions of ion solvation interactions and the mean ionic activity coefficients of lower water content (i.e., ϕ_*w*_ < 0.3) polymers, such as a series of hydrolyzed cross-linked poly­(glycidyl methacrylate)-based materials (referred to as XL – p­(GMA) – z)), where the dielectric constant is reduced relative to XLPEGDA (Figure 2B). Note that details of the application of this modeling approach have been described in a previous publication,^8^ and they are described for the XLPEGDA and XL – p­(GMA) – z polymers in the Supporting Information as applied here (Table S1).

In these low water content polymers (i.e., low dielectric constant polymers), we hypothesized that ionic interactions other than ion solvation interactions (e.g., Coulombic ion–ion interactions such as ion pairing (i.e., the extent of ion dissociation in the hydrated polymer) (Figure 1B) may contribute significantly to mean ionic activity coefficients as well. This hypothesis was motivated by the observation that in aqueous solutions, the extent of ion pairing increases as the dielectric constant of the solvent is reduced.^11,37^ Theoretically, primitive models that approximate the polymer as a dielectric continuum suggest that thermodynamically nonfavorable ion solvation interactions in hydrated polymers can preclude ion partitioning.^38^ Freger recently demonstrated that in the pore model, ion pair formation may be enhanced by thermodynamically nonfavorable ion solvation interactions.^36^ Experimental studies complementing Freger’s theoretical studies would be useful to probe the molecular underpinnings that govern the coupling of ion solvation and ion pairing interactions in hydrated polymers.

In this study, to further understand ion pairing and ion solvation interactions in hydrated polymers and address the discrepancies between our previous application of the pore model (which only included ion solvation interactions) and experimental ionic activity coefficient data for hydrated polymers, we synthesized five series of cross-linked neutral hydrated polymers with varied backbone chemistry (e.g., methacrylate- and styrene/acrylonitrile-based polymers) and functionality (e.g., ether oxygen, hydroxyethyl and hydroxypropyl, and nitrile). The water volume fractions of these polymers ranged from 0.2–0.6, and thus, these values bridge the range of low- and high-water content materials represented by the XL – p­(GMA – z) and XLPEGDA polymers investigated previously. Subsequently, we characterized the dielectric constant and network mesh size properties of the polymers. As discussed previously, cross-linked polymers were chosen because the relative size of the interstitial space between polymer chains (i.e., the characteristic hydrated void space) can be estimated using the polymer network mesh size. Finally, we equilibrated the polymers with 1 M NaCl and characterized their ion sorption properties, which we ultimately used to determine the mean ionic activity coefficients in the polymers.

We report the mean ionic activity coefficients of the polymers as a function of their water content and analyze these data using the classic Born model and pore model. Ultimately, the influence of polymer chemistry on the mean ionic activity coefficients can be rationalized using an application of Freger’s pore model that describes how combinations of ion solvation interactions, which are described using the Born model, and short-range ion–ion interactions, which are described using Bjerrum’s ion association theory, contribute to ionic interactions in hydrated polymers. This modeling approach describes the influence of the network mesh size and dielectric constant of the polymers on the experimentally determined mean ionic activity coefficients with reasonable quantitative accuracy (i.e., within a factor of 2, which is a drastic improvement relative to the classic Born model). These results may be useful to guide engineering strategies for hydrated polymers in a variety of separation and biological applications.

## 2 Theoretical Background

In this section, we outline the relevant theory that describes how electrostatic interactions contribute to mean ionic activity coefficients in hydrated polymers. As discussed in the introduction, the theory described here is adapted from Freger’s pore model.^36^ This model describes a physical picture where free-ions first partition into polymers from the solution and subsequently pair (or associate) in the solvated polymer matrix to minimize the free energy of the ions in their respective electrolyte (i.e., the hydrated polymer or aqueous external solution). Given that generally, ion pair formation is a fast and transient process,^36^ we neglect the direct partitioning of paired ions from solution into the polymer. Note that in the application of these relations to describe ion partitioning in hydrated polymers, the polymer-phase ionic concentrations should be normalized by the volume of water sorbed in the polymer (e.g., units of [mol (ions)/ L­(water sorbed)]).^9,39^ The following analysis is valid for a monovalent (i.e., 1–1) salt, such as NaCl, which is considered here.

### 2.1 Thermodynamic Equilibrium between Polymer and Solution

When a hydrated polymer is initially submerged in an aqueous electrolyte solution (Figure 3A), ions partition from the solution into the polymer to minimize the free energy difference between the two phases (Figure 3B). Effectively, satisfying this equilibrium condition requires that the partial molar free energy of ions in the solution, μ_±_^*s*^, and polymer, μ_±_^*m*^, are equal:

2 $$\mu_{\pm }^{m}=\mu_{\pm }^{s}$$

**Figure 3:**
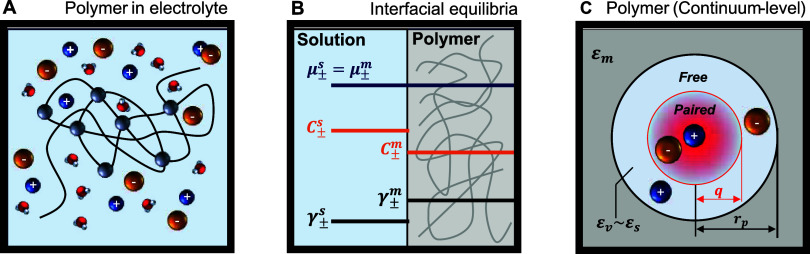
Ion-polymer interactions depicted at (A) the macroscale, (B) the polymer/solution interface, and (C) the continuum-level microscale.

Note that mean ionic quantities for electrolytes are defined such that they contain stoichiometrically weighted contributions from the cation and anion in the electrolyte. For example, for a given electrolyte, *M*_*v*_+__ *X*_*v*_–__, where *v*_+_ and *v*_–_ are the stoichiometric coefficients of the cation and anion in the electrolyte, an arbitrary mean ionic quantity, *g*_±_, is defined as^10^

3 $$g_{\pm }={\left[{{(g}_{+})}^{v_{+}}{\left(g_{-}\right)}^{v_{-}}\right]}^{1/v_{+}+v_{-}}$$

To relate the equilibrium condition to the mean ionic activity coefficients, it is expedient to introduce the definition of mean ionic chemical potential in terms of the mean ionic activity of the electrolyte, *a*_±_^*j*^ as

4 $$\mu_{\pm }^{j}=\mu_{\pm }^{0}+\mathrm{RT}\;\ln\left(a_{\pm }^{j}\right)=\mu_{\pm }^{0}+\mathrm{RT}\;\ln\left(\frac{C_{\pm }^{j}}{C_{\pm }^{0}}\gamma_{\pm }^{j}\right)$$

where *C*_±_^*j*^ is the mean ionic concentration in phase *j*, μ_±_^0^ is the standard chemical potential at the reference concentration *C*_±_^0^, and γ_±_^*j*^ is the mean ionic activity coefficient in phase *j*. For a monovalent salt the mean ionic concentration is effectively equal to the concentration the electrolyte, or salt, *C*_*s*_^*j*^, because *C*_+_^*j*^ = *C*_–_^*j*^ = *C*_*s*_^*j*^.

Combining eqs 2 and 4 results in a thermodynamic expression that describes the mean ionic activity coefficient in the polymer:

5 $$\gamma_{\pm }^{m}=\frac{C_{s}^{s}}{C_{s}^{m}}\gamma_{\pm }^{s}$$

As discussed in the introduction, theoretical models that relate the physical properties and chemical properties of hydrated polymers (e.g., the dielectric constant) to the mean ionic activity coefficient would be useful to understand the molecular underpinnings that govern ionic thermodynamic interactions in polymers. In the subsequent sections, we use theory to derive a model for the mean ionic activity coefficients of ions partitioned in hydrated polymers by considering their self-interactions (Figure 1A) and Bjerrum association interactions (Figure 1B). Longer-range columbic interactions are neglected in this model, which may be reasonable given that ions in hydrated polymers are effectively confined in a low-dielectric constant media such that many ion–ion interactions are effectively “short-range”.

### 2.2 The Pore Model with Ion Solvation Interactions

The dissociation of an ion in a solvent requires the stabilization of its electrostatic charge via the polarization of the solvent molecules.^27^ This electrostatic interaction between ions and their induced polarization charges, or the so-called ion solvation interactions (Figure 1A), can contribute significantly to the mean ionic activity coefficients of dissociated electrolytes.^7,8,29,40^ Classically, the Born model can be used to quantify how ion solvation interactions in an electrolyte solution contribute to the so-called excess solvation energy, *W*_*s*_, by integrating the polarization energy stored in the dielectric medium surrounding a charged ion.^29^ In the classic Born model, the solvent is approximated as a dielectric continuum (i.e., a medium of uniform dielectric constant),^20^ and as discussed in the introduction, this approximation does not apply to hydrated polymers.^8^

In the pore model, the dielectric continuum assumption used to derive the classic Born model is relaxed.^36^ Instead, the model accounts for nanoscale phase segregation between water-rich hydrated void spaces and the low-dielectric constant polymer, and the hydrated polymer is taken as a series of spherical hydrated void spaces (i.e., pores) of the same dielectric constant as the bulk external solution, *ε*_*s*_, dispersed throughout the polymer chains of dielectric constant *ε*_*m*_ (Figure 3C). Although the interstitial hydrated void space between polymer chains is likely randomly distributed and transient due to thermal fluctuations in the polymer chains, the void spaces can be described as spherical pores under the circumstances that the voids are dispersed randomly and lack macro-scale anisotropy, which is reasonable for many hydrated polymers.^29^ The ion is placed in the center of this hydrated void, and under the circumstances that (1) the dielectric constant of the void is approximately equal to that of the external solution, and (2) the characteristic void space is larger than the ion, the excess free energy associated with ion solvation is calculated by integrating the polarization energy stored in the dielectric medium, as^36^

6 $$\frac{\Delta W_{s}}{k_{B}T}=\frac{W_{s}^{m}}{k_{B}T}-\frac{W_{s}^{s}}{k_{B}T}=\frac{e^{2}}{4\pi \varepsilon_{0}k_{B}Tr_{p}}\left(\frac{1}{\varepsilon_{m}}-\frac{1}{\varepsilon_{s}}\right)=A$$

where *k*_B_ *T* is the molecular thermal energy, *r*_*p*_ is the characteristic hydrated void space, *e* is the elementary charge, *z*_+_ and *z*_–_ are the valences of the cation and anion, respectively (i.e., 1 for NaCl), and *ε*_0_ is the vacuum permittivity. Note that eq 6 calculates Δ*W*_*s*_, which is defined here as the difference in the total excess solvation energy of salt in the polymer, *W*_*s*_^*m*^, and the external solution, *W*_*s*_^*s*^, which effectively serves as a reference state. A key result of eq 6 is that, given the approximations discussed previously, all terms describing the influence of ionic size on the excess solvation energy difference are negligible in comparison to those describing the influence of the void size on the excess solvation energy.^8,29,36^ Equation 6 can be simplified by introducing the Bjerrum lengths in the polymer and solution, λ_*m*_ and λ_*s*_, respectively, which are defined generally as

7 $$\lambda_{j}=\frac{e^{2}}{4\pi \varepsilon_{0}k_{B}T\varepsilon_{j}}$$

and defining the characteristic ratio *A*:

8 $$A=\frac{\lambda_{m}-\lambda_{s}}{r_{p}}$$

This characteristic ratio effectively characterizes the difference in the solvation energy of an ion in the void space of a hydrated polymer relative to its value in the bulk.

The mean ionic activity coefficient of the free (i.e., fully dissociated) ions in the polymer can be obtained directly from the excess solvation energy, which can be used to approximate the partial molar mean ionic Gibbs free energy of the electrolyte (i.e., Δ*G̅*_±_^*E, j*^/*RT* ≈ Δ*W*_*s*_/2*k*_B_ *T*). Therefore, combining eq 6 with eq 1 and eq 4 results in the following expression for the mean ionic activity coefficients:

9 $$\gamma_{\pm ,f}^{m}=\exp\left[\frac{A}{2}\right]\gamma_{\pm ,f}^{s}$$

where the subscript *f* is introduced to denote the free ions, which will be used to distinguish the associated (or paired) ions that will be introduced in the subsequent section. Equation 9, which is referred to herein as the pore model with ion solvation interactions, effectively describes the influence of ion solvation interactions on the mean ionic activity coefficients (Figure 1). In the simplest case where ion pairing interactions are negligible, eq 9 can be used to model the mean ionic activity coefficient of ions partitioned in a hydrated polymer, and therefore, combining eq 9 with eq 5 results in an expression that describes the concentration of free ions that initially partition into the polymer from the aqueous electrolyte as

10 $$C_{s,f}^{m}=C_{s,f}^{s}\;\exp\left[-\frac{A}{2}\right]$$

### 2.3 The Pore Model with Ion Solvation and Ion Pairing Interactions

In many electrolyte solutions, ions do not fully dissociate (i.e., ions can exist as associated, or paired, species that have reduced electrostatic charge relative to fully dissociated species).^41^ Ion pairing interactions, which are generally considered to be thermodynamically favorable interactions, effectively contribute to stabilizing charge separation. It is hypothesized that ion pairing contributes to ionic interactions in hydrated polymers as well as in aqueous electrolytes.^12,24,36,38,42^

Classically, Bjerrum proposed a strategy to describe the influence of ion pairing interactions on mean ionic activity coefficients, where the effective activity coefficients in an electrolyte are obtained by modification of the free (i.e., dissociated) ionic activity coefficient:^21^

11 $${\gamma_{\pm }^{j}=\left(1-\alpha^{j}\right)\gamma }_{\pm ,f}^{j}$$

In eq 11, α^*j*^ is the degree of association, or the fraction of paired ions in phase *j*, which is defined as

12 $$\alpha^{j}=\frac{C_{s,p}^{j}}{C_{s,p}^{j}+C_{s,f}^{j}}$$

where *C*_*s,p*_^*j*^ is the concentration of the paired ions in phase *j*. Effectively, eqs 11 and 12 suggest that in Bjerrum’s approach, information about the concentration of paired and free ions in the electrolyte is required to account for the influence of ion pairing interactions on the mean ionic activity coefficients in electrolytes.

The concentrations of paired and free ions in an electrolyte solution can be related using the association equilibria of a monovalent salt, *MX*:^36,41^

13 $$M^{+}+X^{-}↔\mathrm{MX}$$

which is described by the equilibrium concentration quotient as described in more detail by Marcus and Hefter, *K*_*p*_^*j*^, as^36,41^

14 $$K_{p}^{j}=\frac{C_{p}^{j}}{C_{+}^{j}C_{-}^{j}}=\frac{C_{s,p}^{j}}{{{(C}_{s,f}^{j})}^{2}}$$

Bjerrum suggested that the pairing constant can be calculated by performing an integration of the Boltzmann factor, exp­[−*U*(*x*)/*k*_*B*_ *T*], over all paired states.^21^ Bjerrum proposed that ions are considered paired if they are separated by a distance *x* that is less than the cutoff distance, *q*, which separates free and paired states (Figure 1B). Therefore, the pairing constant can be calculated as^36^

15 $$K_{p}^{j}=N_{A}\int_{a}^{q}\exp\left[\frac{-U^{j}\left(x\right)}{k_{B}T}\right]4\pi x^{2}dx$$

where *a* is the distance of closest approach of the ion. For oppositely charged ions in solution, the interion potential in the Boltzmann factor, *U* ^*j*^(*x*) is defined as^36^

16 $$U^{j}\left(x\right)=-z_{+}z_{-}\left[\left(\frac{e^{2}}{4\pi \varepsilon_{0}\varepsilon_{s}x}\right)+W_{s}^{j}\left(x\right)\right]$$

Therefore, for ions partitioned in the hydrated polymer, the interion potential can be defined using the solution as a reference state:

17 $$U^{m}\left(x\right)=U^{s}\left(x\right)-z_{+}z_{-}\Delta W_{s}$$

If the solvation energy is approximated to be uniform in the hydrated void space of the polymer, which is reasonable in the spherical-pore approximation,^43^ and the space of the hydrated void is larger than the cutoff distance (i.e., *r*_*p*_ > *q*) (Figure 3C), the pairing constant in the polymer can effectively be calculated as a function of that in solution:

18 $$K_{p}^{m}=N_{A}\int_{a}^{q}\exp\left[\frac{-U^{s}\left(x\right)}{k_{B}T}+\Delta W_{s}\right]4\pi x^{2}dx=\exp\left[A\right]K_{p}^{s}$$

where the exponential factor in eq 18 effectively describes how large values of the solvation energy difference between solution and polymer contribute to increasing the probability of ion pair formation in the polymer relative to the solution. Combining eq 18, the mass action law (written for both the polymer and solution) (eq 13), the definition of the degree of association (written for both the polymer and solution) (eq 12), and the expression derived for the concentration of free salt in the polymer (eq 10), results in the following expression for the degree of association in the polymer:

19 $$\alpha^{m}=\frac{\alpha^{s}}{\alpha^{s}+\exp[-A/2]\left(1-\alpha^{s}\right)}$$

Equations 19 and 9 can be substituted into eq 11 and rearranged to obtain the final expression for the mean ionic activity coefficient that accounts for both ion association and ion solvation interactions:

20 $$\gamma_{\pm }^{m}=\frac{\gamma_{\pm }^{s}}{\alpha^{s}+\exp[-A/2]\left(1-\alpha^{s}\right)}$$

This model, referred to herein as the pore model with ion solvation and pairing interactions (Figure 3C), can be applied directly from experimental data given the degree of association of the electrolyte and the mean ionic activity coefficient in the external solvent, the polymer dielectric constant, and a measurement of the characteristic size of the hydrated void. The solution properties (i.e., the degree of association and mean ionic activity coefficients) are tabulated for many aqueous electrolytes. In the subsequent section, we highlight the experimental techniques used to synthesize the polymers considered in this report and characterize their dielectric constant and network mesh size, which is used as a proxy for the size of the hydrated void.

## 3 Experimental Section

### 3.1 Materials

Five series of cross-linked polymer films were synthesized using free radical-initiated polymerization (Table 1). As discussed in the introduction, cross-linked polymers were chosen so that the network mesh size could be used to estimate quantitatively the characteristic hydrated void space of the polymers, which contributes to the mean ionic activity coefficients (Section 2). Additionally, the synthetic matrix of materials was chosen to represent materials with a wide range of water content, dielectric constant, and network mesh size. The relevant compositions of the prepolymerization mixtures used to cross-link the polymer films are reported in Table 1.

**Table 1:**
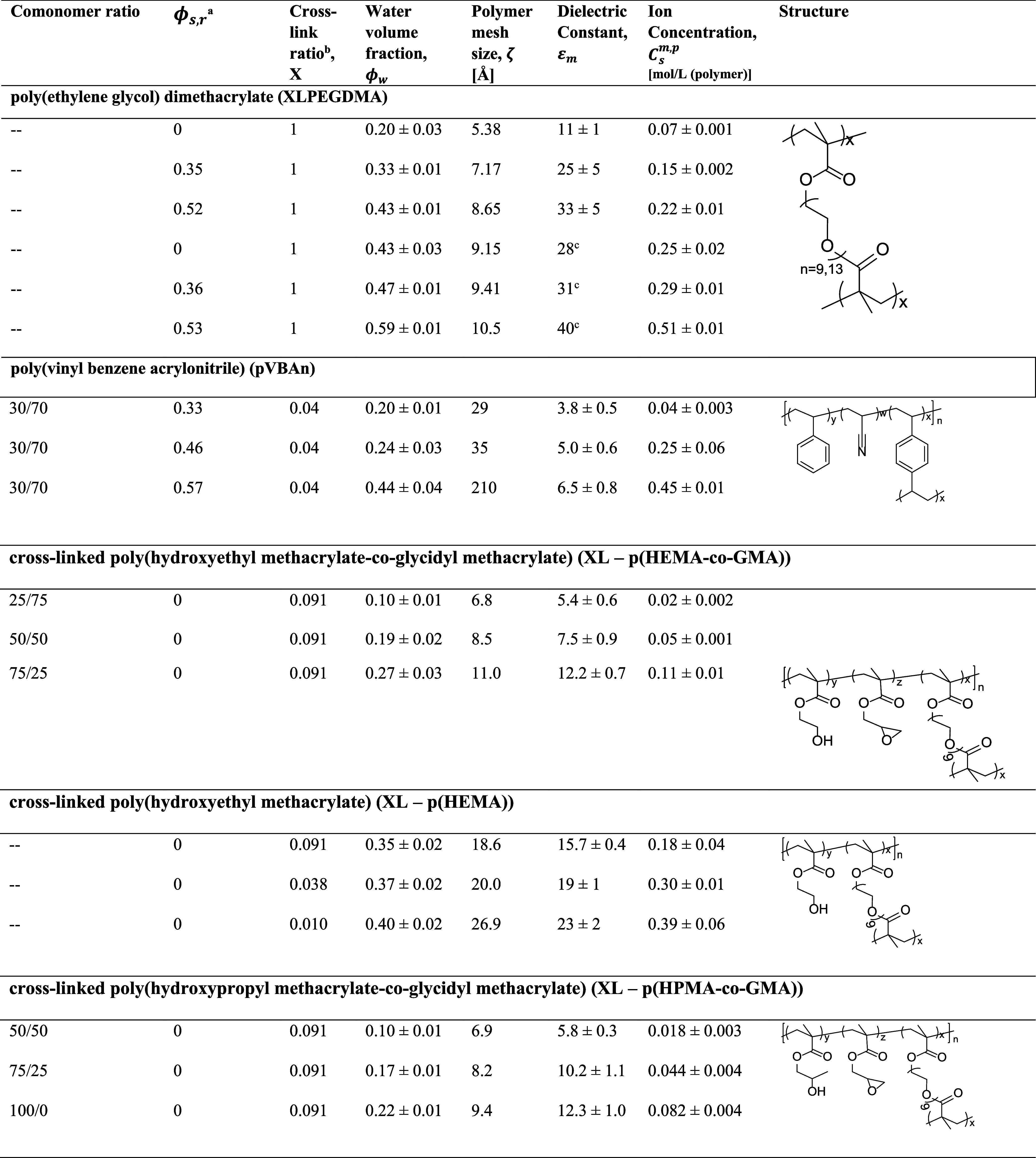
Materials Considered in This Study

a Volume fraction of solvent in the prepolymerization mixture.

b Mass ratio of cross-linker and comonomers, in units of [g (cross-linker)/g (cross-linker and vinyl monomers)].

c Polymer films were too fragile to sustain the wrapping procedure required for the dielectric relaxation spectroscopy measurement, and the dielectric constant was estimated using the Maxwell – Garnett model.

All polymer films were synthesized according to previously reported free-radical initiated polymerization procedures.^44–47^ Generally, the prepolymerization solutions, which contained an aliquot of a free-radical initiator (which was either hydrocyclohexyl phenyl ketone (HCPK) at 0.1 wt % for XLPEGDMA, HCPK at 1 wt % for XL – p­(HEMA), XL – p­(HEMA-*co*-GMA), and XL – p­(HPMA-*co*-GMA), or benzoin ethyl ether (BEE) for p­(VBAN), were mixed until homogenized. For the XLPEGDMA and p­(VBAN) films, a solvent, which was either water or DMSO for XLPEGDMA or p­(VBAN), respectively, was added to the prepolymerization solutions at a volume fraction of ϕ_*s*,*r*_ to facilitate the homogenization of the prepolymerization solutions.

These homogeneous solutions were then sandwiched between two quartz plates separated by spacers that were used to control the film thickness, which were then placed in a UV-cross-linking chamber (Spectroline, Select Series), and irradiated with 120 μJ/cm^2^ of 312 nm light. The time scale of this irradiation process, which was used to facilitate the decomposition of the radical initiator and simultaneously polymerize and cross-link the monomers in the solutions, was either 90 (XLPEGDMA), 600 (XL-p­(HEMA-*co*-GMA), XL – p­(HEMA), and XL – p­(HPMA-*co*-GMA)), or 3600 (p­(VBAn)) seconds. Afterward, the quartz plates were removed from the cross-linking chamber and separated, and the polymerized films were peeled and submerged in deionized water. All polymers were stored for at least 48 h before use, and over this period the water was changed at least twice. For the p­(VBAn) polymers, which were cross-linked in the presence of dimethyl sulfoxide (DMSO), the polymers were boiled in deionized water for 48 h to facilitate the exchange of water for dimethyl sulfoxide.

### 3.2 Water Content

The water content of the polymers was determined using a previously reported hydrostatic weighing technique. The water-swollen polymers were removed from the solution, quickly blotted dry to facilitate the removal of excess surface water, and weighed to determine their hydrated mass, *m*_*wet*_. The samples were then dried under vacuum at 80 °C for at least 48 h, and then subsequently weighed to obtain their dry mass, *m*_*dry*_. The water uptake, *w*_*u*_, of the samples was then calculated as^28^

21 $$w_{u}=\frac{m_{\mathrm{wet}}-m_{\mathrm{dry}}}{m_{\mathrm{dry}}}$$

After the dry mass of the polymers was obtained, the polymers were submerged in an auxiliary solvent that does not readily partition into the polymers (e.g., cyclohexane) and weighed. The dry density was calculated from Archimedes’ principle as^28^

22 $$\rho_{p}=\frac{m_{\mathrm{aux}}}{m_{\mathrm{aux}}-m_{\mathrm{dry}}}\left(\rho_{\mathrm{aux}}-\rho_{\mathrm{air}}\right)$$

where *m*_aux_ is the mass of the auxiliary solvent, and ρ_aux_ and ρ_air_ are the dry densities of cyclohexane and air, respectively. Finally, the volume fraction of water in the polymer, ϕ_*w*_, was calculated using a volume additivity assumption:^28^

23 $$\phi_{w}=\frac{w_{u}}{w_{u}+\rho_{w}/\rho_{p}}$$

where ρ_*w*_ is the density of water.

The network mesh size was calculated using the number-average molecular weight between cross-links, *M̅*_*c*_, which is determined from an extension of the Flory–Rehner and Peppas-Merril equations, as developed by Lucht and Peppas to describe isotropic, highly cross-linked polymer networks that were initially cross-linked in the presence of a diluent:^48^

24 $$\frac{1}{{\mathrm{M̅}}_{c}}=\frac{2}{{\mathrm{M̅}}_{n}}-\frac{\frac{1}{\rho_{p}V_{1}}\left[\ln\left(1-\phi_{p}\right)+\phi_{p}+\chi \phi_{p}^{2}\right]\left[1-\frac{1}{N}{\left(\frac{\phi_{p}}{\phi_{p,r}}\right)}^{2/3}\right]}{\phi_{p,r}\left({\left(\frac{\phi_{p}}{\phi_{p,r}}\right)}^{1/3}-\frac{1}{2}\left(\frac{\phi_{p}}{\phi_{p,r}}\right)\right){\left(1+\frac{1}{N}{\left(\frac{\phi_{p}}{\phi_{p,r}}\right)}^{1/3}\right)}^{2}}$$

*N* represents the number of links of the polymer chain, which is calculated as^48^

25 $$N=\lambda {\mathrm{M̅}}_{c}/M_{r}$$

where λ is the polymer backbone bond factor and *M*_*r*_ is the molecular weight of the repeat unit between the cross-links. In eq 24, *M̅*_*n*_ is the number-average molecular weight of the polymer chains prior to cross-linking, ϕ_*p,r*_ is the volume fraction of polymer in the relaxed state (i.e., before cross-linking such that ϕ_*p, r*_ = 1–ϕ_*s,r*_), ϕ_*p*_ is the polymer volume fraction in the polymer after cross-linking and swollen in water (i.e., ϕ_*p*_ = 1 – ϕ_*w*_), and χ is the polymer–solvent interaction parameter (i.e., the so-called Flory–Huggins interaction parameter). In the application of eq 24 to polymer networks that are prepared under conditions where the chain growth occurs concurrent with cross-link formation, because it is impossible to measure *M̅*_*n*_ before cross-linking, it is common to assume that the conversion of the monomers is sufficient such that the term 2/*M̅*_*n*_ is small and can be neglected in eq 24.^49,50^ Once the number-average molecular weight between cross-links is determined, the root-mean-square end-to-end distance of the polymer chains can be calculated as^49^

26 $${\left({\mathrm{r̅}}_{0}^{2}\right)}^{1/2}={\left(C_{n}Nl^{2}\right)}^{1/2}$$

where *l* is the average bond length along the backbone of the repeat unit (i.e., 1.54 Å for C–C bonds, as is relevant for all the materials considered here). Finally, the mesh size is obtained directly using the root-mean-square end-to-end distance as^48^

27 $$\zeta =\phi_{p}^{-1/3}{\left({\mathrm{r̅}}_{0}^{2}\right)}^{1/2}$$

### 3.3 Dielectric Relaxation Spectroscopy (Dielectric Constant and State of Water)

Microwave dielectric relaxation spectroscopy (DRS) was used to characterize the frequency dependent relative permittivity of the polymers.^7,28,31,51,52^ Hydrated polymers were subjected to an oscillating electromagnetic field in the microwave frequency range (45 MHz – 26.5 GHz), which was generated by a Keysight N9928A vector network analyzer (VNA). A 5 cm long, 3.5 mm diameter coaxial transmission line was used as the sample holder, and this line was connected to the VNA using shielded coaxial cables. The VNA collected signals of the electromagnetic radiation reflected from and transmitted through the polymer samples. These signals were expressed as four scattering parameters, which were related to the relative complex permittivity of the samples using a previously described mathematical procedure.^28^

The geometry of the sample holder in the measurement requires that the hydrated polymer samples fill the annular space of the coaxial sample holder. Generally, to fill this annulus, hydrated polymer films were cut into 0.5 cm strips and quickly wrapped tightly around the inner conductor until enough polymer was wrapped to completely fill the annular space of the sample holder. The time scale of this wrapping procedure and the subsequent measurement was consistently shorter than 2 min, and over this time scale we verified that a negligible amount of water evaporated from the samples using a static weighing measurement concurrent with the time scale of the DRS measurement procedure. For the hydrated XLPEGDMA films, which did not have the mechanical properties to sustain the wrapping procedure, dry polymer films were used to fill the annulus and subsequently hydrated by submerging the entire sample holder in deionized water. After hydration, care was taken to remove excess water by quickly purging the sample holder with air.

The frequency-dependent complex relative permittivity obtained from this measurement was fit to a two-parameter Havrilak-Negami model, as^52^

28 $$\varepsilon^{*}=\varepsilon_{\infty }+\sum_{j=1}^{2}\frac{\Delta \varepsilon_{j}}{{\left[1+{\left(i\omega \tau_{j}\right)}^{1-\alpha_{j}}\right]}^{\beta_{j}}}$$

where Δ*ε*_*j*_ and τ_*j*_ are the dielectric strength and characteristic time scale, respectively, of the *j*th relaxation, ε_∞_ is the high-frequency static permittivity, ω is the angular frequency, and α_*j*_ and β_*j*_, are empirically determined shape parameters (the best fit for the spectra obtained here were obtained using α_*j*_ = β_*j*_ = 1, which is a special case of the Havrilak-Negami model referred to as an ideal Debye relaxation). The dielectric constant is calculated form these fitted parameters as the sum of the high frequency permittivity and the dielectric strength of the rotationally bound and bulk-like water dielectric relaxation processes as^52^

29 $$\varepsilon_{m}=\varepsilon_{\infty }+\Delta \varepsilon_{\mathrm{RB}}+\Delta \varepsilon_{\mathrm{BL}}$$

The model was constrained so that the first relaxation process corresponded to a bulk-like water relaxation (i.e., τ_1_ = 8.8 ps),^31^ and that the second relaxation corresponded to a restricted water molecule relaxation (i.e., τ_2_ > 8.8 ps), which implies a three population model for the state of water in the polymer (i.e., water that relaxes at the same time scale as the bulk is considered bulk-like (BL) water, water that relaxes time scales greater than the bulk is referred to as rotationally bound (RB) water, and water that does not relax (i.e., is invisible to the DRS measurement) is referred to as irrotationally bound (IB) water). The concentration of each of these water molecule populations was determined by combining a mass-balance on the total concentration of water in the polymer, *c*_*w*_^*m*^:

30 $$c_{w}^{m}=c_{w,\mathrm{BL}}^{m}+c_{w,\mathrm{RB}}^{m}+c_{w,\mathrm{IB}}^{m}$$

with an application of the Kirkwood-Froelich equation that relates the concentration of water in each state to the dielectric strength of the relaxation process corresponding to that state, as^53^

31 $$c_{w,j}^{m,\mathrm{ap}}=c_{w}^{s}F_{\mathrm{KF}}\frac{2\varepsilon_{m}+\varepsilon_{\infty }}{\varepsilon_{m}}\Delta \varepsilon_{j}$$

In eqs 30 and 31, *c*_*w,j*_^*m*^ corresponds to the concentration of water in state *j* and *c*_*w*_^*s*^ is the concentration of water in the external solution. The superscript *ap* is used to denote that eq 31 is calculated by normalizing the Kirkwood – Froehlich Equation written for a specific relaxation process to that of deionized water, using the function *F*_KF_, which is related to the relative permittivity properties of deionized water as^53^

32 $$F_{\mathrm{KF}}=\frac{\varepsilon_{s}}{\left(\varepsilon_{s}-\varepsilon_{\infty }^{s}\right)\left(2\varepsilon_{s}+\varepsilon_{\infty }^{s}\right)}$$

where ε_*s*_ and ε_∞_^*s*^ are the low and high frequency limits of the relative permittivity of deionized water. It is important to note that in this analysis, we assume that only water molecules contribute to the dipolar relaxation process in the solvated polymer, which is reasonable given generally, the dielectric constant of dry polymers is significantly less than that of water.

### 3.4 Mean Ionic Activity Coefficients

The mean ionic activity coefficients were determined experimentally by measuring the equilibrium concentration of ions that partitioned into the polymer from a solution of known concentration. This data was then used to calculate the mean ionic activity coefficients according to eq 5. Note that for 1 M NaCl, the mean ionic activity coefficient in the solution is equal to 0.67, as determined using the Pitzer model (i.e., *C*_*s*_^*s*^ = 1 mol/L and γ_±_^*s*^ = 0.67).^17^

The equilibrium ion concentration that partitioned into the polymer from the sodium chloride solution was characterized using a kinetic desorption technique.^45^ The polymer samples were first equilibrated in 1 M NaCl for 48 h. These equilibration solutions were changed at least twice to facilitate the equilibration of the polymer/solution mixtures. After this procedure, the polymers were removed from the electrolyte solution, quickly wiped to remove surface solution droplets, and their thickness and diameter were measured using digital calipers. Next, the polymers were placed in a desorption solution containing 60 mL of deionized water in a temperature controlled jacketed vessel. The desorption solution was stirred to mitigate boundary layer effects, and the temperature was held constant at 25 °C.

The time-dependent conductivity and temperature of the desorption solution was measured using a conductivity meter (Cond 7310, WTW). As salt desorbed from the polymer, the conductivity of the solution increased, and the conductivity was measured until it stabilized to a constant value. The concentration of salt in the desorption solution was determined from this stabilized solution conductivity using a calibration curve. The relationship between conductivity and concentration is temperature dependent, so the calibration curve data was measured at the experimental temperature (25 ± 0.1 °C).

The concentration of NaCl was calculated from the long-time desorption data as^27,54,55^

33 $$C_{s}^{m,p}=\frac{C_{d}V_{d}}{V_{p}}$$

where *C*_*s*_^*m,p*^ is the concentration of salt in the polymer in units of [mol/L (swollen polymer)], *C*_*d*_ is the salt concentration of the desorption solution calculated from the long-time desorption solution conductivity using the calibration curve, *V*_*d*_ is the volume of the desorption solution, and *V*_*p*_ is the volume of the swollen polymer, which can be geometrically determined by measuring the thickness and surface area of a sample before the salt extraction process. To convert the experimentally determined salt concentration to units of [mol/L (water sorbed)], which are relevant for thermodynamic analysis, the polymer-basis concentration is divided by the water volume fraction:

34 $$C_{s}^{m}=\frac{C_{s}^{m,p}}{\phi_{w}}$$

## 4 Results and Discussion

### 4.1 Polymer Properties and Mean Ionic Activity Coefficients

Here, we report the experimentally determined network mesh size, dielectric constant, and mean ionic activity coefficients of the hydrated polymers considered in this study as a function of water content. As discussed in the introduction, polymer water content significantly contributes to the thermodynamic environment experienced by ions in the hydrated polymer. The water content values are reported in Table 1.

Here, the equilibrium water content of the hydrated polymer networks was effectively controlled by three synthetic variables: (1) the prepolymerization solvent content, ϕ_*s, r*_, (i.e., the volume fraction of water or dimethyl sulfoxide used for the preparation of XLPEGDMA and p­(VBAn), respectively), (2) the ratio of hydrophilic and hydrophobic comonomers incorporated in the prepolymerization mixture (i.e., the ratio of HEMA to GMA or HPMA to GMA in XL – p­(HEMA-*co*-GMA) or XL – p­(HPMA-*co*-GMA), respectively), and (3) the amount of cross-linker incorporated in the prepolymerization mixture, which is related to the cross-linking ratio, X, and was varied in XL – p­(HEMA) (Table 1). Generally, water content increases as the prepolymerization solvent content increases, the water content increases as the ratio of the hydrophilic comonomer increases, and the water content increases as the cross-linking ratio is reduced (Table 1). Through control of these synthetic variables, the polymers synthesized in this study had water volume fractions ranging from approximately 0.1 to 0.6 (Table 1).

#### 4.1.1 Network Mesh Size

The parameters used to obtain the network mesh size from the water content data are reported in Table 2. For many of the polymers, these parameters were taken as their values for similarly structured polymers reported elsewhere.^49,56,57^ However, for the p­(VBAn) polymers, no such data were available for the Flory–Huggins parameter, so for these materials the parameter was estimated using an application of the Flory–Huggins model for liquid water swelling that is commonly used to estimate the water–polymer interaction parameter for desalination polymers (Table 2).^27^ This approximation may be reasonable for the p­(VBAn) materials in particular given they are the least cross-linked of all the materials synthesized in this study (i.e., there is relatively less elastic force present in the p­(VBAn) network relative to the more densely cross-linked films (e.g., XLPEGDMA)) and has been used to estimate the Flory–Huggins parameters in dilute networks previously.^50^

**Table 2:** Parameters Used for the Calculation of the Polymer Mesh Size

material	Flory–Huggins interaction parameter, χ	Flory’s characteristic ratio, *C*_*n*_
hydroxyl-containing methacrylates^a^	χ = 0.32 + 0.904ϕ_*p*_	7
XLPEGDMA^56,57^	χ = 0.28 + 0.892ϕ_*p*_	4
p(VBAn)	χ ≈ [− ln(1–ϕ_*p*_) – ϕ_*p*_]/ϕ_*p*_^2^	10

a Data obtained for XL – p­(HEMA), XL – p­(HEMA-*co*-GMA), and XL – p­(HPMA-*co*-GMA), as reported for HEMA-containing polymers reported by Peppas et al.^49^ Note that the relationship for the hydration-dependent Flory–Huggins parameter of these materials was obtained from direct regression of the data in Table 1.^49^

Generally, the network mesh size of the hydrated polymers increases with increasing water volume fraction (Figure 4). This scaling relationship between the water content and network mesh size is expected based on the definition of the network mesh size (eq 27) and is commonly reported in the literature.^48,49,57,58^ These results are consistent with a physical picture where, as the polymer water content increases, the network swells to facilitate the increase in the network mesh size.

**Figure 4:**
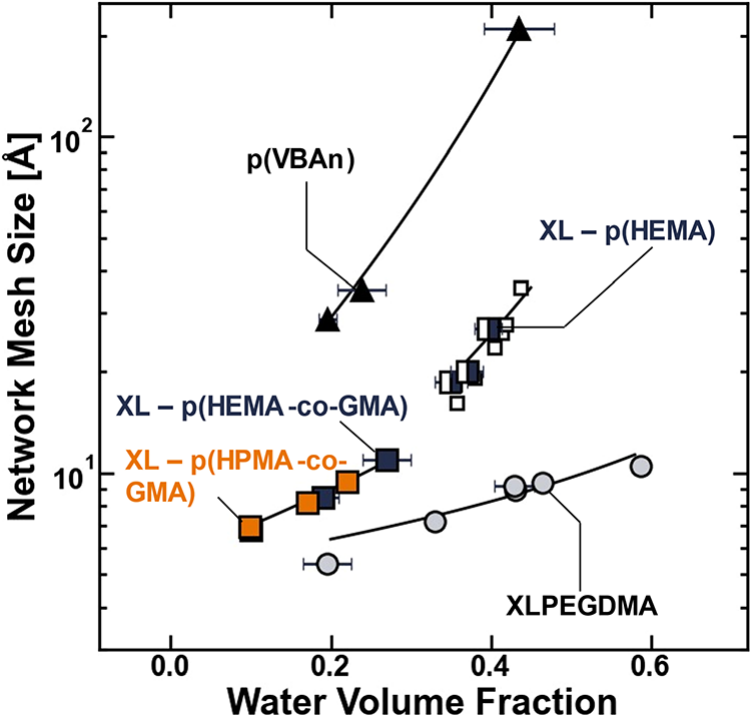
(A) Network mesh size plotted as a function of the water volume fraction for XLPEGDMA (gray circle solid), p­(VBAn) (▲), XL – p­(HEMA-*co*-GMA) (blue box solid), XL – p­(HEMA) synthesized here (◨), XL – p­(HEMA) reported by Richbourg and Peppas (□)^48^ (for reference), or XL – p­(HPMA-*co*-GMA) (orange box solid). The lines were fit to the experimental data using Canal and Peppas’ empirical power law, ζ = *a*(1 – ϕ_*w*_)^−*n*^, where *a* and *n* are empirical parameters.^58^

Previously, Canal and Peppas presented a power-law correlation that suggests the network mesh size scales with the inverse polymer volume fraction (i.e., ζ ∼ (1 – ϕ_*w*_)^−*n*^, where *n* is an arbitrary empirical parameter).^58^ This power law was fit to the hydration-dependent mesh size data for each polymer to describe the functional form of the scaling relationship observed between the network mesh size and water volume fraction (Figure 4). These empirical scaling relationships, which are useful in the development of models that relate the mean ionic activity coefficients to the water volume fraction of the polymer, are provided in Table 3.

**Table 3:** Hydration-Dependent Network Mesh Size and Dielectric Constant

**material**	**network mesh size**, ***ζ*** **[Å]**	**dielectric constant**, **ε**_***m***_
XL – p(HEMA-*co*-GMA)	ζ = 5.6(1 – ϕ_*w*_)^−2.1^	$\left(1-\phi_{w}\right)\frac{\varepsilon_{\mathrm{pMMA}}-\varepsilon_{m}}{\varepsilon_{\mathrm{pMMA}}+2\varepsilon_{m}}=\phi_{w}\left(\frac{\varepsilon_{w}-\varepsilon_{m}}{\varepsilon_{w}+{2\varepsilon }_{m}}\right)$
XL – p(HEMA)	ζ = 3.9(1–ϕ_*w*_)^−3.7^	$\left(1-\phi_{w}\right)\frac{\varepsilon_{\mathrm{pMMA}}-\varepsilon_{m}}{\varepsilon_{\mathrm{pMMA}}+2\varepsilon_{m}}=\phi_{w}\left(\frac{\varepsilon_{w}-\varepsilon_{m}}{\varepsilon_{w}+{2\varepsilon }_{m}}\right)$
XL p(HPMA-*co*-GMA)	ζ = 5.6(1–ϕ_*w*_)^−2.1^	$\frac{\varepsilon_{m}-\varepsilon_{w}}{\varepsilon_{m}+{2\varepsilon }_{w}}={(1-\phi }_{w})\left(\frac{\varepsilon_{\mathrm{pMMA}}-\varepsilon_{w}}{\varepsilon_{\mathrm{pMMA}}+{2\varepsilon }_{w}}\right)$
XLPEGDMA	ζ = 5.2(1–ϕ_*w*_)^−0.9^	$\frac{\varepsilon_{m}-\varepsilon_{w}}{\varepsilon_{m}+{2\varepsilon }_{w}}={(1-\phi }_{w})\left(\frac{\varepsilon_{\mathrm{pMMA}}-\varepsilon_{w}}{\varepsilon_{\mathrm{pMMA}}+{2\varepsilon }_{w}}\right)$
p(VBAn)	ζ = 8.3(1–ϕ_*w*_)^−5.6^	$\frac{\varepsilon_{m}-\varepsilon_{\mathrm{pS}}}{\varepsilon_{m}+{2\varepsilon }_{\mathrm{pS}}}=\phi_{w}\left(\frac{\varepsilon_{w}-\varepsilon_{\mathrm{pS}}}{\varepsilon_{w}+{2\varepsilon }_{\mathrm{pS}}}\right)$

The scaling relationship between the water content and network mesh size is different for each polymer (Figure 4), and this result suggests that specific elements of polymer chemistry, and not just the water content alone, contribute to the network mesh size. In the simplest explanation, the chemistry-dependent scatter in the polymer network mesh size data about a given water content can be rationalized using a discussion of Flory’s characteristic ratio *C*_*n*_. Flory’s characteristic ratio is effectively a measurement of chain stiffness that describes the proportionality between the root-mean-square end-to-end distance of a chain and the number of links in the chain (eq 26).^48^ Therefore, when Flory’s characteristic ratio is small, flexible chains tend to entangle such that they are less elongated than stiff chains that can form fewer entanglements. In other words, at a given number of links in a polymer chain, the root-mean-square end-to-end distance of a polymer chain is reduced as Flory’s characteristic ratio decreases, and this phenomenon contributes to reducing the network mesh size at a given water content as the Flory characteristic ratio decreases (c.f. Table 2 and Figure 4).

#### 4.1.2 Hydrated Polymer Dielectric Constant

As discussed in the methods, the dielectric constant of the hydrated polymers was obtained from the frequency-dependent complex relative permittivity spectra, which, for brevity, are provided in the Supporting Information (Figure S1). Like the network mesh size, the dielectric constant of the hydrated polymers increases with increasing water volume fraction (Figure 5), which is expected based on previous reports in the literature. These results are consistent with a physical picture where, as the number of polar water molecules in the polymer increases, the capacity of the molecules that constitute the polymer network to polarize and store energy in the presence of an applied electromagnetic field increases as well.

**Figure 5:**
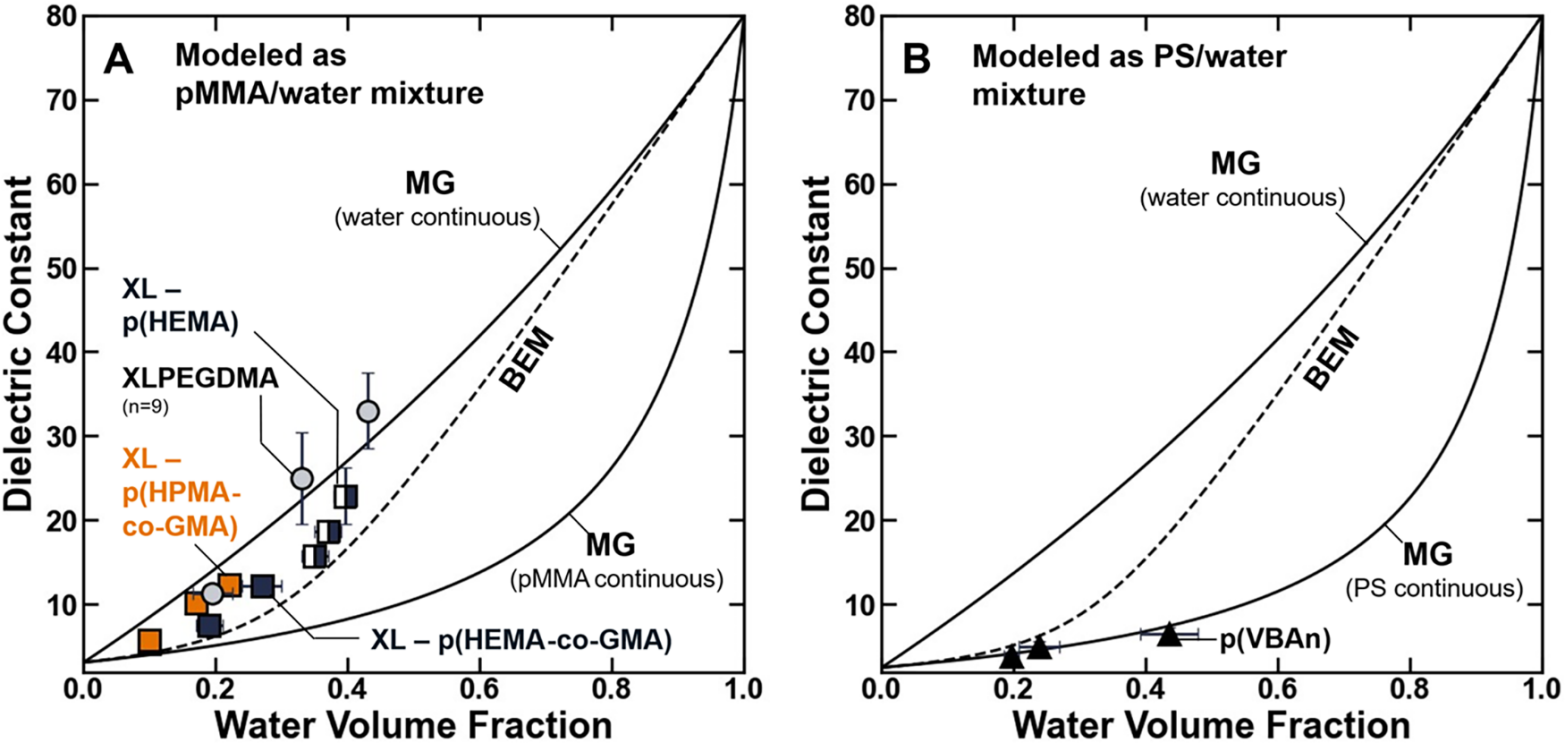
Polymer dielectric constant plotted as a function of the water volume fraction for (A) XLPEGDMA (gray circle solid), XL – p­(HEMA-*co*-GMA) (blue box solid), XL – p­(HEMA) (◨), or XL – p­(HPMA-*co*-GMA) (orange box solid), and (B) p­(VBAn) (▲). The solid lines are plotted using either the polymer or water-continuous applications of the Maxwell – Garnett model (MG), and the dashed line is plotted using the Bruggeman Effective Medium (BEM) approximation. The uncertanties were taken as the standard deviation from the mean of three measurements.

One strategy to describe this hydration-dependence of the polymer dielectric constant is to use heterogeneous phase models that relate the dielectric constant of a heterogeneous material to its composition and the dielectric constant of the pure components.^59^ The Maxwell-Garnett model (MG), which is one such modeling approach, has been applied successfully to describe the dielectric constant of low water content (ϕ_*w*_ < 0.3) hydrated polymers.^60^ The Maxwell – Garnett model approximates the heterogeneous material with a morphology where the disperse phase is distributed throughout the continuous phase as a series of spherical inclusions. The dielectric constant of the heterogeneous material, (which, for the polymer, is ε_*m*_) is calculated as a function of the dielectric constants of the continuous and disperse phases, ε_1_ and ε_2_, respectively, as^59^

35 $$\frac{\varepsilon_{m}-\varepsilon_{1}}{\varepsilon_{m}+{2\varepsilon }_{1}}=\phi_{2}\left(\frac{\varepsilon_{2}-\varepsilon_{1}}{\varepsilon_{2}+{2\varepsilon }_{1}}\right)$$

where ϕ_2_ is the volume fraction of the disperse phase. The Maxwell – Garnett model can be applied to hydrated polymer materials with either the polymer or water taken as the continuous phase (Figure 5). Here, the model was applied using values of 78 for the dielectric constant of water, and the “pure” (i.e., dry) polymer dielectric constant was approximated as its value for dry poly­(methyl methacrylate) (i.e., ε_pMMA_ = 3.12) for the methacrylate-based polymers (Figure 5A), or its value for poly­(styrene) (i.e., ε_PS_ = 2.5) for the styrene/acrylonitrile-based polymers (Figure 5B).^27^

For most of the XLPEGDMA, XL – p­(HPMA-*co*-GMA), XL – p­(HEMA), and p­(VBAn) polymers, the Maxwell – Garnett model generally described accurately the functional form of the observed scaling relationship between the water content and dielectric constant of the hydrated polymers (Figure 5). Visually, the model described XLPEGDMA and XL – p­(HPMA-*co*-GMA) when water was taken as the continuous phase (Figure 5A), and the model described the p­(VBAn) polymers when polystyrene was taken as the continuous phase (Figure 5B). However, the dielectric constants of XL – p­(HEMA-*co*-GMA) and XL – p­(HEMA) were generally between the predictions of the two applications of the model (Figure 5A), and for these polymers, alternative to the Maxwell – Garnett model, the hydration-dependent dielectric constant properties are better described by the Bruggeman effective medium (BEM) approximation:^59^

$$\left(1-\phi_{2}\right)\frac{\varepsilon_{1}-\varepsilon_{m}}{\varepsilon_{1}+{2\varepsilon }_{m}}=-\phi_{2}\left(\frac{\varepsilon_{2}-\varepsilon_{m}}{\varepsilon_{2}+{2\varepsilon }_{m}}\right)$$ 36

which is another heterogeneous phase-modeling approach that accounts for the transition between disperse and continuous phases with increasing volume fraction of the disperse phase. For convenience, the model that best describes each set of experimental water content/dielectric constant data is summarized in Table 3.

These observations highlight that more than just compositional factors alone govern whether a material is continuous or disperse in either phase. For example, although the water phase is relatively dilute in XLPEGDMA and XL – p­(HPMA-*co*-GMA) (i.e., ϕ_*w*_ < 0.5), the dielectric constant properties of the polymers are most accurately described by an application of the MG model where water is taken as the continuous phase (Figure 5). Similar observations have been reported where the dilute phase of a polymer exhibits continuous phase behavior (e.g., nitrogen/oxygen permeability properties of PTMSP/PPP blends).^61^ Ultimately, these observations suggest that other elements of polymer chemistry (e.g., specific water/polymer interactions or morphological factors) contribute to the hydrated polymer dielectric constant as well as its composition (i.e., the water volume fraction).

#### 4.1.3 Mean Ionic Activity Coefficients

The mean ionic activity coefficients in the polymer are larger than the mean ionic activity coefficients in the solution (Figure 6). This observation suggests that the excess partial molar Gibbs free energy associated with ionic interactions in the polymer is larger than that in the external solution, and under these circumstances, ionic interactions are thermodynamically nonfavorable in the polymer relative to the solution (eq 1). As the water content of the polymers increases, the mean ionic activity coefficients reduce to approach their value in the aqueous electrolyte (Figure 6), suggesting that thermodynamic ionic interactions become increasingly favorable in the polymer as the water content increases. To a first approximation, these observations are reasonable, because it is expected that, in a hypothetical polymer with a water volume fraction of 1, ionic interactions would be identical to those in the external solution.

**Figure 6:**
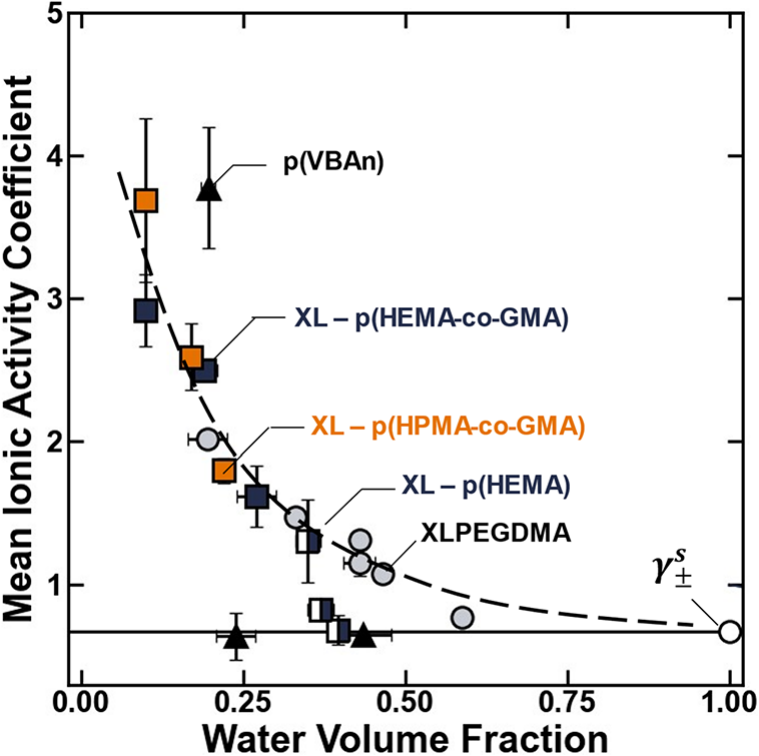
Mean ionic activity coefficients in the polymer plotted as a function of the water volume fraction for XLPEGDMA (gray circle solid), p­(VBAn) (▲), XL – p­(HEMA-*co*-GMA) (blue box solid), XL – p­(HEMA) (◨), and XL – p­(HPMA-*co*-GMA) (orange box solid). The empty circle (○) represents a hypothetical membrane of pure electrolyte where the mean ionic activity coefficient is expected to be equal to its value in the external solution. The dashed line is drawn to guide the eye, and the solid line is drawn to represent the situation where the mean ionic activity coefficients in the polymer are the same as those in the external solution. The uncertanties were taken as the standard deviation from the mean of three measurements.

At a given water content, there is generally some chemistry-dependent scatter in the mean ionic activity coefficient data, although interestingly, the scatter is somewhat reduced relative to that in the network mesh size and dielectric constant data (c.f. Figures 4, 5, and 6). Within the context of the pore model, it is expected that these mean ionic activity coefficients are governed by a combination of the characteristic hydrated void space (i.e., the network mesh size) and the polymer dielectric constant (eq 20). In the subsequent section, we analyze the mean ionic activity coefficient data within the context of the pore model to rationalize the chemistry-dependent scatter in the hydration-dependent activity coefficient data.

### 4.2 Modeling

Within the context of the pore model (Section 2), variations in the network mesh size and dielectric constant contribute to the mean ionic activity coefficients in a hydrated polymer. Effectively, these polymer properties influence the energy required to solvate dissociated ions (i.e., electrostatically charged species) that are partitioned in a polymer (eq 6). Additionally, these polymer properties influence the degree of ion pairing (eq 19), and within this application of the pore model, ion-pairing is a phenomenon that effectively contributes to lowering the ion–ion interaction energy when two ions move into the same pore.

In the model, as the polymer dielectric constant is reduced relative to its value in the bulk, the magnitude of the difference in the solvation energy between ions partitioned in the hydrated void space of a polymer and the external bulk aqueous solution increases (Figure 7A). The magnitude of the excess solvation energy also increases as the size of the characteristic hydrated void space is reduced (Figure 7A). These conditions (i.e., reduced dielectric constant and size of the hydrated void space) effectively correspond to a physical picture where ions are effectively confined near a low dielectric constant medium. Under these circumstances, there are few polar molecules that can contribute to stabilizing the electrostatic potential of the ions in the polymer, which contributes to increasing their energy state relative to ions in the bulk solution (i.e., the solvation free energy is large and positive) (Figure 7A). Note that the combined influence of the characteristic hydrated void space and dielectric constant on ionic interactions in the polymer matrix can be summarized through the characteristic electrostatic ratio *A*, which is inversely proportional to the void space size and dielectric constant and effectively describes the difference in the solvation energy in the membrane relative to in a bulk electrolyte (eq 8).

**Figure 7:**
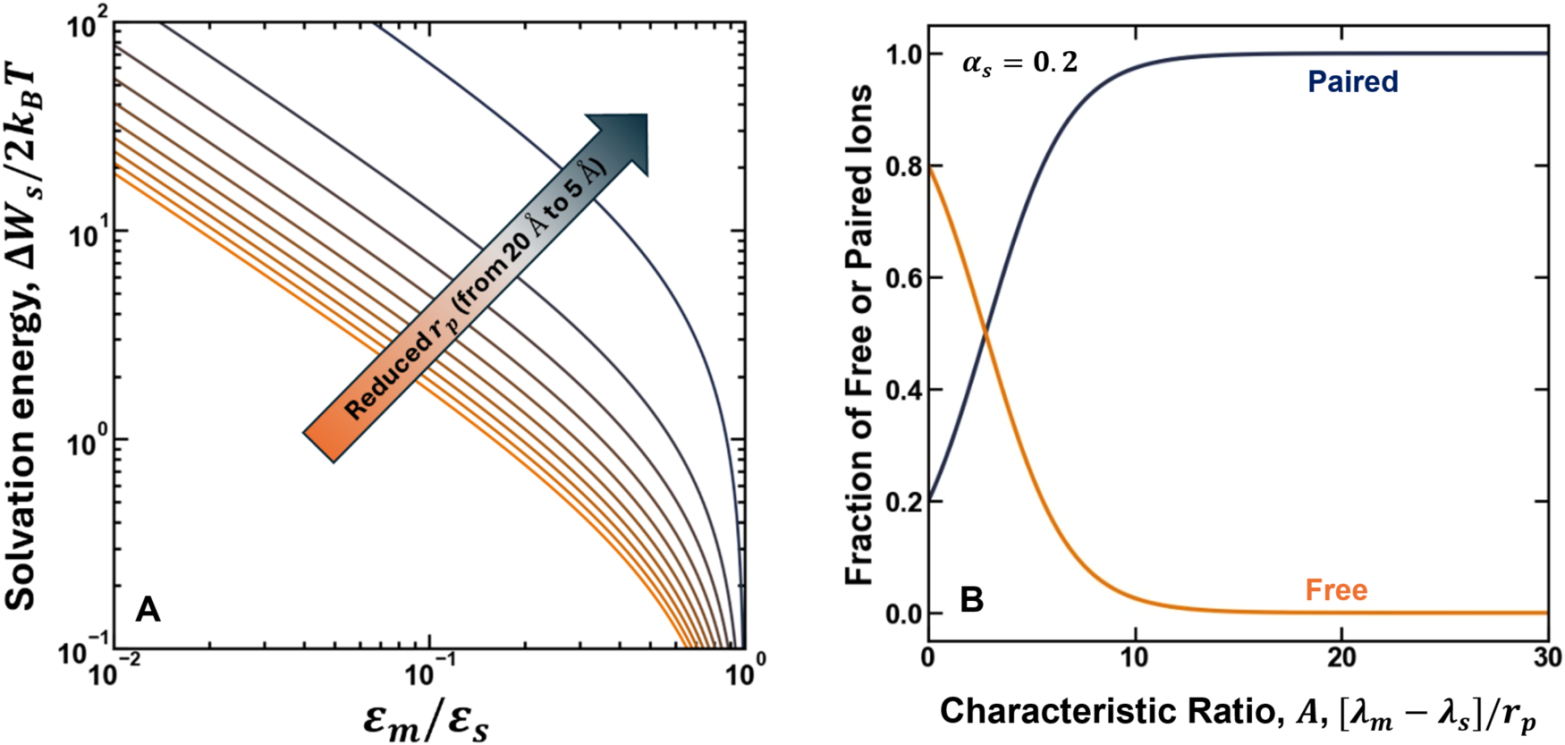
Theoretical description of the pore model that demonstrates how the dielectric constant and network mesh size influence ionic interactions in a hydrated polymer. In (A) the solvation energy is plotted as a function of the solution-normalized dielectric constant (eq 6), and the color gradient is used to illustrate the variation in the characteristic hydrated void space (i.e., from orange lines to blue lines represents a reduction in the void space from 20 Å to 5 Å). In (B), the fraction of free or paired ions in the polymer is plotted as a function of the characteristic ratio, *A*, that describes the magnitude of the excess solvation energy difference between solution and polymer.

As the characteristic ratio, *A*, increases, the increased energy state of ions in the polymer (relative to those in the bulk solution) increases the propensity of free ions to pair in the membrane relative to the solution. Effectively, ion-pair formation contributes to reducing the interion potential of the dissociated ions, which in turn reduces their excess free energy (eq 11). As a result, the fraction of ions in the polymer that exist in paired states increases significantly as the characteristic ratio increases (Figure 7B). Because neutral ion-pairs are relatively thermodynamically ideal species relative to charged dissociated ions, the increased degree of ion-pairing in the polymer at high values of *A* effectively reduces the effective mean ionic activity coefficients in the polymer relative to what is expected under the circumstance where all the ions in the polymer exist as dissociated species (eq 11).

#### 4.2.1 Application of the Pore Model to Describe Neutral Polymers and Justification of Assumptions

The pore model can be applied to the polymers in this study by calculating the characteristic ratio, *A*, directly from the experimental data. Once the experimentally determined value of *A* is obtained, and given the degree of association of ions in the external solution (here, for 1 M NaCl, α_*s*_ was taken approximately as 0.2 as reported by Sujanani et al.)^12^ the pore model can be used directly to calculate the mean ionic activity coefficient in the polymer using either eq 20, which includes both ion solvation and ion pairing interactions, or using eq 6, which excludes ion-pairing interactions and is consistent with the previous application of the pore model^8^ represented in Figure 2. Here, the dielectric constant is obtained directly from the dielectric relaxation spectroscopy (or, for the XLPEGDMA polymers with n = 13 that were too fragile to undergo the wrapping procedure required for the DRS measurement, estimated using the Maxwell – Garnett model (Table 3)) and the characteristic void space is estimated using the polymer network mesh size (i.e., *r*_*p*_ ∼ ζ) (Table 1).

Alternatively, the pore model can be used to predict the hydration-dependent mean ionic activity coefficients given an appropriate relationship for the hydration-dependent network mesh size and dielectric constant. As discussed in Section 4.1, Canal and Peppas’ empirical power law^58^ can be used to describe the relationship between the mesh size and water content, and various applications of heterogeneous phase models can be used to describe the relationship between the dielectric constant and water content (Table 3). By substituting these relationships into the pore model (again, either eq 20 or eq 6 to represent the model with and without ion-pairing interactions, respectively), a theoretical prediction of the mean ionic activity coefficients as a function of the water content is obtained. For the sake of brevity, comparison between these applications of the hydration-dependent models and experimental data are provided in the Supporting Information (Figure S2).

Effectively, three assumptions were used in the application of the pore model that is employed here. First, a key assumption in the model development is that the polymer can be approximated as a heterogeneous dielectric that contains distinct hydrated void spaces where the dielectric constant is comparable to that of the bulk. The physical picture described by the pore model can be verified using state-of-water data that is obtained during the dielectric relaxation spectroscopy measurement, which provides information about the concentration of water in the polymer that has similar dipolar properties as water in the bulk (Section 3.3). Generally, in each polymer, the fractional concentration of this bulk-like water (i.e., water that has the same time scale of dipolar motions relative to water in the bulk) is nonzero (Figure 8), and this results support the physical picture proposed in the pore model where the polymer contains distinct hydrated void spaces where the molecular motions of water are not influenced by interactions with the polymer.

**Figure 8:**
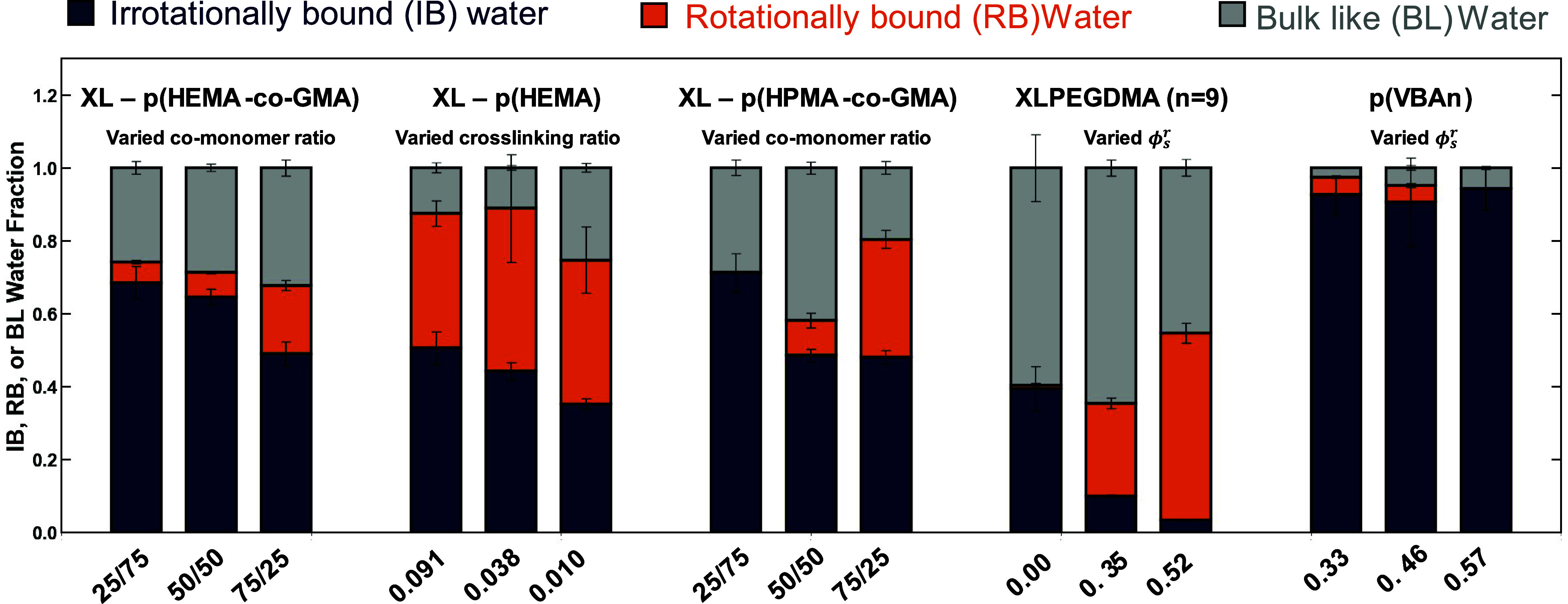
Fractional concentration of irrotationally bound water in the polymer (i.e., *c*_*w, IB*_^*m*^/*c*_*w*_^*m*^) for XLPEGDMA, p­(VBAn), XL – p­(HEMA-*co*-GMA), XL – p­(HPMA-*co*-GMA), and XL – p­(HEMA). Note that the *x* axis labels correspond to the ratio of HEMA/GMA or HPMA/GMA in the prepolymerization solution for XL – p­(HEMA-*co*-GMA) or XL-p­(HPMA-*co*-GMA), respectively, the cross-linking ratio for XL – p­(HEMA), and the prepolymerization solvent volume fraction for XLPEGDMA and p­(VBAn). The standard deviation was calculated using standard error propagation of the dielectric constant and water uptake data.

Next, also in the derivation of the solvation energy, we applied an approximation that required the characteristic void space to be larger than the ionic radius (eq 6), which for sodium and chloride is approximately 1–2 Å.^33^ Generally, the network mesh sizes ranged from 5–200 Å (Figure 4), and thus this approximation is also reasonable for these materials. Finally, to derive the expression for the pairing constant in the polymer, we applied an approximation that required the characteristic hydrated void to be larger than the cutoff distance that separates paired and free states (eq 19). In the hydrated void space, given the dielectric constant is approximated as that in the solution, the cutoff distance (calculated using Bjerrum’s original approach) is approximately 3.5 Å.^36^ Therefore, this approximation is reasonable as well for the materials considered in this report.

#### 4.2.2 Influence of Network Mesh Size and Dielectric Constant on Mean Ionic Activity Coefficients

As discussed previously, the combined influence of the network mesh size and dielectric constant on the mean ionic activity coefficients is described through Freger’s characteristic ratio *A*, which scales with the network mesh size and dielectric constant as *A* ∼ 1/ε_*m*_ζ (eq 11). This ratio *A* decreases exponentially to approach zero as the water volume fraction of the polymer increases and approaches unity (Figure 9), which corresponds to the observations that the dielectric constant and network mesh size increase as the water content approaches unity (Figures 4 and 5). Effectively, given that *A* corresponds to the magnitude of the excess solvation energy difference between the solution and polymer (eq 11), this observation corresponds to a physical picture where, as polymer hydration increases, the thermodynamic environment experienced by ions in the polymer becomes increasingly comparable to that in the external solution.

**Figure 9:**
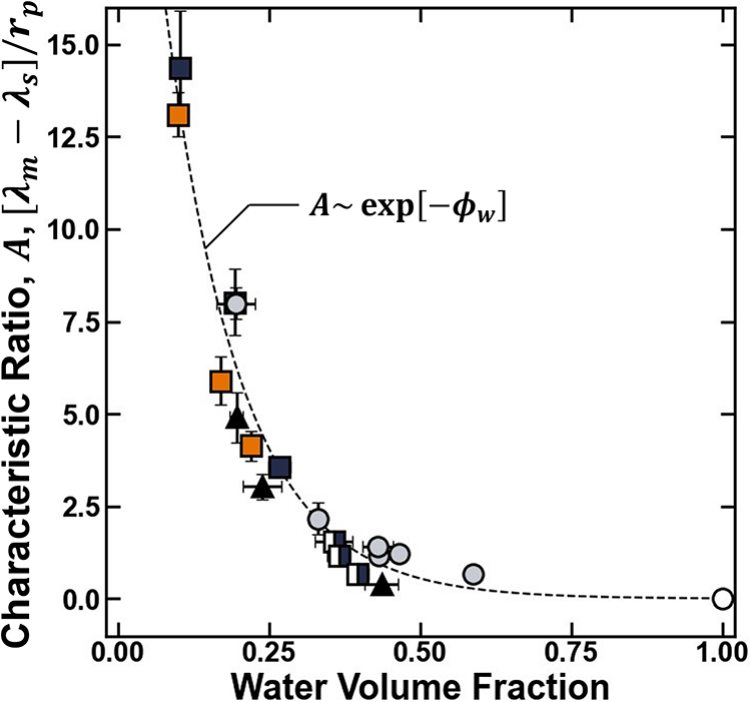
Characteristic ratio *A* of the polymer plotted as a function of the water volume fraction for XLPEGDMA (gray circle solid), p­(VBAn) (▲), XL – p­(HEMA-*co*-GMA) (blue box solid), XL – p­(HEMA) (◨), and XL – p­(HPMA-*co*-GMA) (orange box solid). The empty circle (○) represents a hypothetical membrane of pure electrolyte where characteristic ratio is zero (i.e., the Bjerrum lengths in the solution and membrane are equal). The dashed line is plotted to guide the eye and the uncertanties were taken as the standard deviation from the mean of three measurements.

The mean ionic activity coefficients of the polymer increase as the characteristic ratio increases (Figure 10). In other words, the mean ionic activity coefficients increase as the dielectric constant and network mesh size decrease. These results are consistent with the physical picture proposed by the pore model (i.e., the results are described qualitatively by the pore model (c.f. Figures 7 and 10).

**Figure 10:**
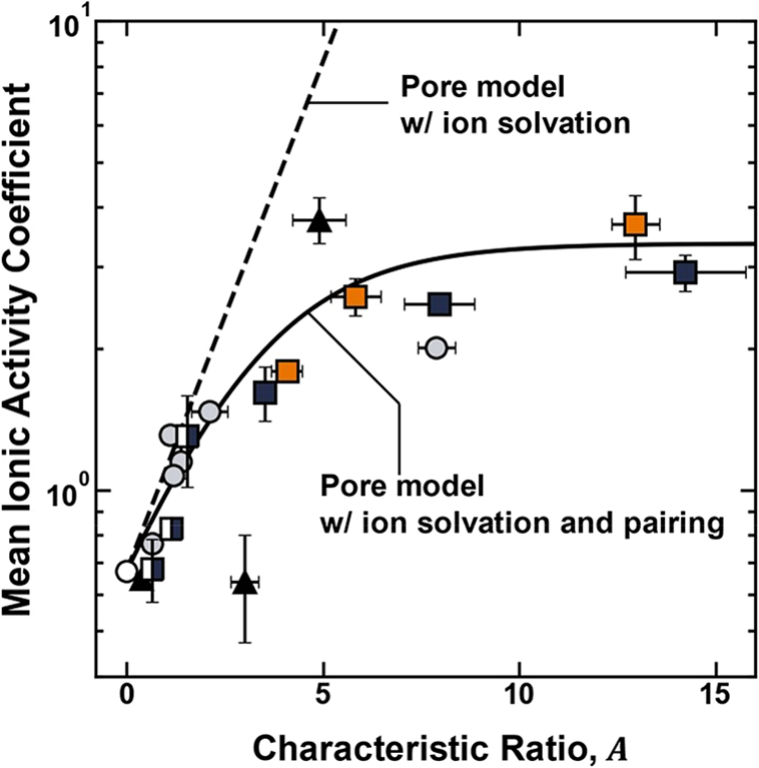
Mean ionic activity coefficient of the polymer plotted as a function of the characteristic ratio for XLPEGDMA (gray circle solid), p­(VBAn) (▲), XL – p­(HEMA-*co*-GMA) (blue box solid), XL – p­(HEMA) (◨), and XL – p­(HPMA-*co*-GMA) (orange box solid). The dashed line is plotted using the pore model without ion pairing (eq 9) and the solid line is plotted using the pore model with ion pairing (eq 20). The uncertanties were taken as the standard deviation from the mean of three measurements.

Generally, the visual agreement between the model and experimental data increases significantly when ion-pairing interactions are included, and the discrepancies between the model applications with and without ion-pairing interactions are more significant when the characteristic ratio is large e.g., *A* > 3, (Figure 10). The point where the two model applications diverge generally corresponds to the point where the theoretically determined fraction of paired ions in the polymer exceeds the theoretical fraction of free ions (c.f. Figures 7B and 10). These results suggest that the extent of ion-pairing in the neutral polymer matrix may be reasonably controlled through modifications of the network mesh size and dielectric constant (i.e., to increase the degree of association in the polymer, one could either reduce the network mesh size or reduce the dielectric constant) and suggest that ion pairing interactions are important for modeling mean ionic activity coefficients in polymers with combinations of low dielectric constant and small network mesh size.

The predictions of the pore model with ion-pairing interactions generally describes the experimental mean ionic activity coefficients of the data within a factor of 2 (Figure 11). This agreement between the model and experimental data represents a marked improvement relative to classic thermodynamic models that have been used to describe thermodynamic interactions in hydrated polymers (i.e., the classic Born model applied with the dielectric continuum assumption) which often have discrepancies with experimental data of several orders of magnitude (Figure 2). These results highlight potential predictive uses of the pore model to describe the influence of the polymer dielectric constant and network mesh size on thermodynamic interactions in hydrated polymers *a priori*.

**Figure 11:**
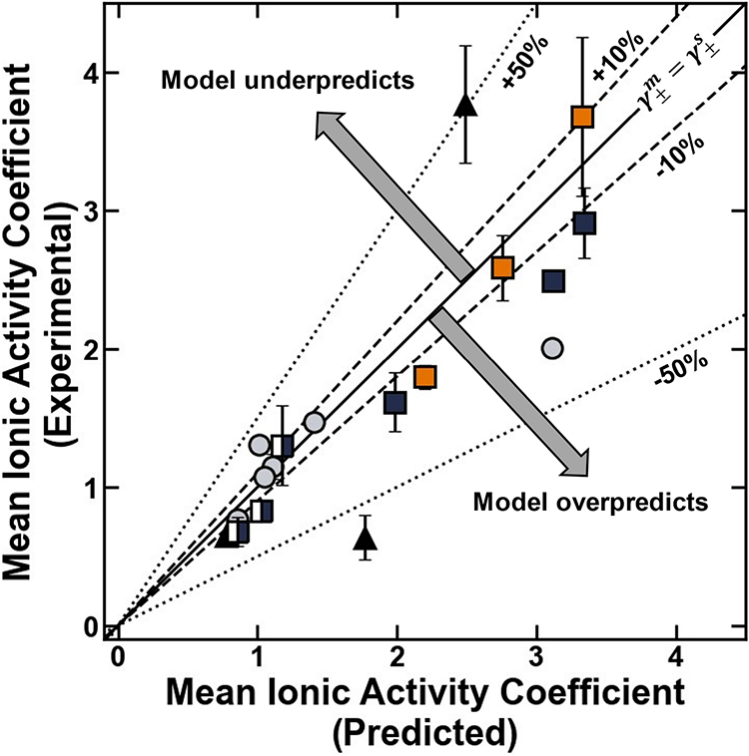
Experimentally determined mean ionic activity coefficients plotted as a function of the theoretically predicted mean ionic activity coefficients using the pore model with ion pairing and ion solvation interactions (eq 20) for XLPEGDMA (gray circle solid), p­(VBAn) (▲), XL – p­(HEMA-*co*-GMA) (blue box solid), XL – p­(HEMA) (◨), and XL – p­(HPMA-*co*-GMA) (orange box solid). The solid line is drawn to represent parity between the model and experiment, the dashed lines represent agreement between the model and experiment within 10%, and the dotted lines represent agreement between the model and experiment within 50%. The uncertanties were taken as the standard deviation from the mean of three measurements.

In the materials where the pore model prediction differs from the experimental data, the model tends to overpredict the mean ionic activity coefficients (Figure 11). One hypothesis that explains these discrepancies is that associative interactions between ions and polar functionalities tethered to the polymer can also contribute to the mean ionic activity coefficients of the polymers. For example, electronegative functionalities, such as ether oxygen, which are contained in many of the polymers considered here, have been shown to have strong associative interactions with alkali earth metals such as sodium,^62–64^ and these ion-polymer interactions may contribute to the mean ionic activity coefficients in a similar manner as the ion–ion pairing interactions.

## 5 Conclusions

We determined the mean ionic activity coefficients for sodium chloride in five series of neutral cross-linked hydrated polymers containing varied backbone chemistry, functionality, and degree cross-linking. We characterized the polymer network mesh size, dielectric constant, and mean ionic activity coefficients in the hydrated polymers, and we reported these properties as a function of the polymer water volume fraction. Generally, as the water content of the polymers increases, the network mesh size and dielectric constant increase, and the mean ionic activity coefficients decrease. These results generally imply that ionic thermodynamic interactions in the hydrated polymer become increasingly thermodynamically favorable as the water content, and thus the dielectric constant and network mesh size, increases.

To rationalize these results, we used Freger’s pore model to derive an expression that describes ion solvation and ion pairing interactions in hydrated polymers and relates the network mesh size and polymer dielectric constant to the mean ionic activity coefficients in neutral hydrated polymers. After verifying the approximations used to derive the pore model using the experimental data, we demonstrated that the application of the model accounting for both ion pairing and ion solvation interactions, which requires information about the characteristic hydrated void space (taken here, for cross-linked polymers, as the network mesh size) and dielectric constant of the hydrated polymer, described more accurately the mean ionic activity coefficients in the polymer relative to the application of the pore model that accounted for ion solvation interactions alone. These results suggest that ion pairing interactions are important for modeling mean ionic activity coefficients in hydrated polymers. Additionally, these results provide a theoretical basis to describe the manner that polymer properties contribute to ionic interactions in hydrated neutral polymers. Ultimately, these results may be useful to model ionic interactions and inform engineering strategies for hydrated polymer membrane materials.

## Supplementary Material



## Notes

Information about models used in Figure 1, frequency-dependent relative permittivity, hydration-dependent mean ionic activity coefficient models, parameters used in the model application and resulting output (PDF)
