# Supplementary material for: The Influence of Ion Solvation and Association Interactions on Mean Ionic Activity Coefficients in Neutral Polymeric Membranes
Source: Macromolecules. 2025 Nov 5;58(22):12388–403. doi: 10.1021/acs.macromol.5c01588 (PMC12659442; doi:10.1021/acs.macromol.5c01588)
Supplement: Supplementary file 1 [file ma5c01588_si_001.pdf]

Supporting Information

The influence of ion solvation and association interactions on mean ionic activity coefficients in neutral polymeric membranes

*Sean M. Bannon,[1] Rachel L. Fetter,[1] Viatcheslav Freger,[2] and Geoffrey M. Geise[1,]**

*To whom correspondence should be addressed: geise@virginia.edu

[1]Department of Chemical Engineering, University of Virginia, 385 McCormick Road, Charlottesville, Virginia 22903 USA

[2]Wolfson Department of Chemical Engineering, Technion - Israel Institute of Technology, Haifa, 32000, Israel

Table S1. Hydration-dependent network mesh size and dielectric constant data used to model the mean ionic activity coefficients of XLPEGDA and XL – p(GMA) – z as reported in Figure 2. For the pore model with ion solvation, both these relationships were substituted into Eq. 6 in the main text with $r_p = \zeta(\phi_w)$, and for the Classic Born model, the Maxwell – Garnett model for the dielectric constant was substituted into Eq. 6 in the main text with $r_p = r_s$, which is the mean ionic cavity radii that is approximately equal to 0.18 Å for NaCl. The values of the dielectric constants of water, ε_w , and the dry methacrylate-based polymers, ε_{pMMA}, were taken as 78 and 3.12 as discussed in the main text. Note that differences in the network mesh size for the XLPEGDA films as calculated here and those reported previously result from subtle differences in the equations used to calculate the network mesh size (for consistency in this report, the approach outlined in Section 3.2 was applied to these polymers to directly calculate the network mesh size).

**Material**	**Network mesh size, ζ [Å]**	**Dielectric constant, ε_m**
XLPEGDA	$\zeta = 3.2(1-\phi_w)^{-1.3}$	$\frac{\varepsilon_m-\varepsilon_w}{\varepsilon_m+2\varepsilon_w} = (1-\phi_w)\left(\frac{\varepsilon_{pMMA}-\varepsilon_w}{\varepsilon_{pMMA}+2\varepsilon_w}\right)$
XL – p(GMA) – z	$\zeta = 3.9(1-\phi_w)^{-2.6}$	$\frac{\varepsilon_m-\varepsilon_{pMMA}}{\varepsilon_m+2\varepsilon_{pMMA}} = \phi_w\left(\frac{\varepsilon_w-\varepsilon_{pMMA}}{\varepsilon_w+2\varepsilon_{pMMA}}\right)$

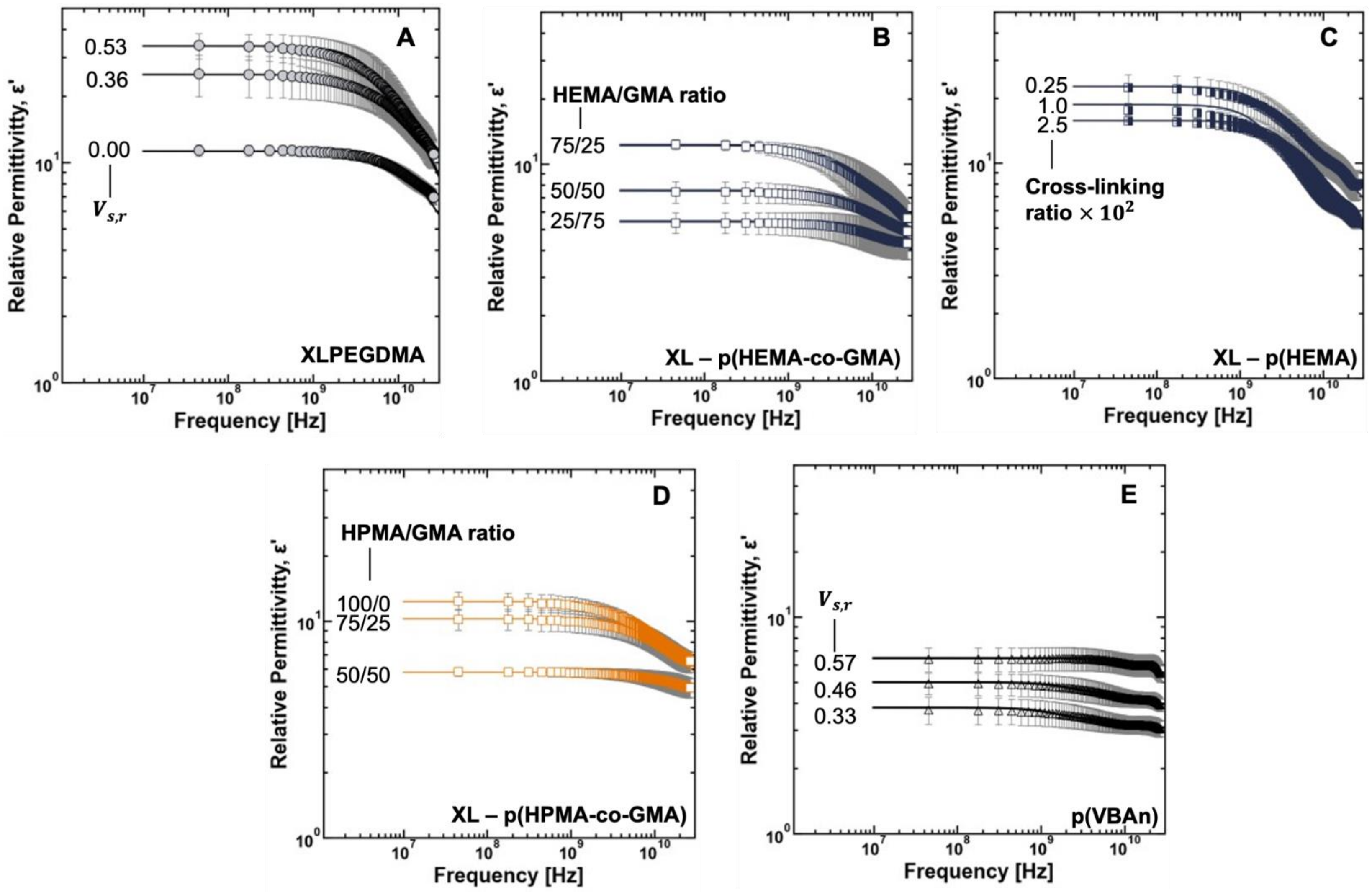

Figure S1. The frequency-dependent real part of the complex relative permittivity for (A) XLPEGDMA (●), (B) XL – p(HEMA-co-GMA) (■), (C) XL – p(HEMA) (◨), (D) XL – p(HPMA-co-GMA) (■), and (E) p(VBAn) (▲). The lines correspond to the two-parameter Havriliak-Negami model that was fit to the complex and real parts of the complex relative permittivity as discussed in the main text.

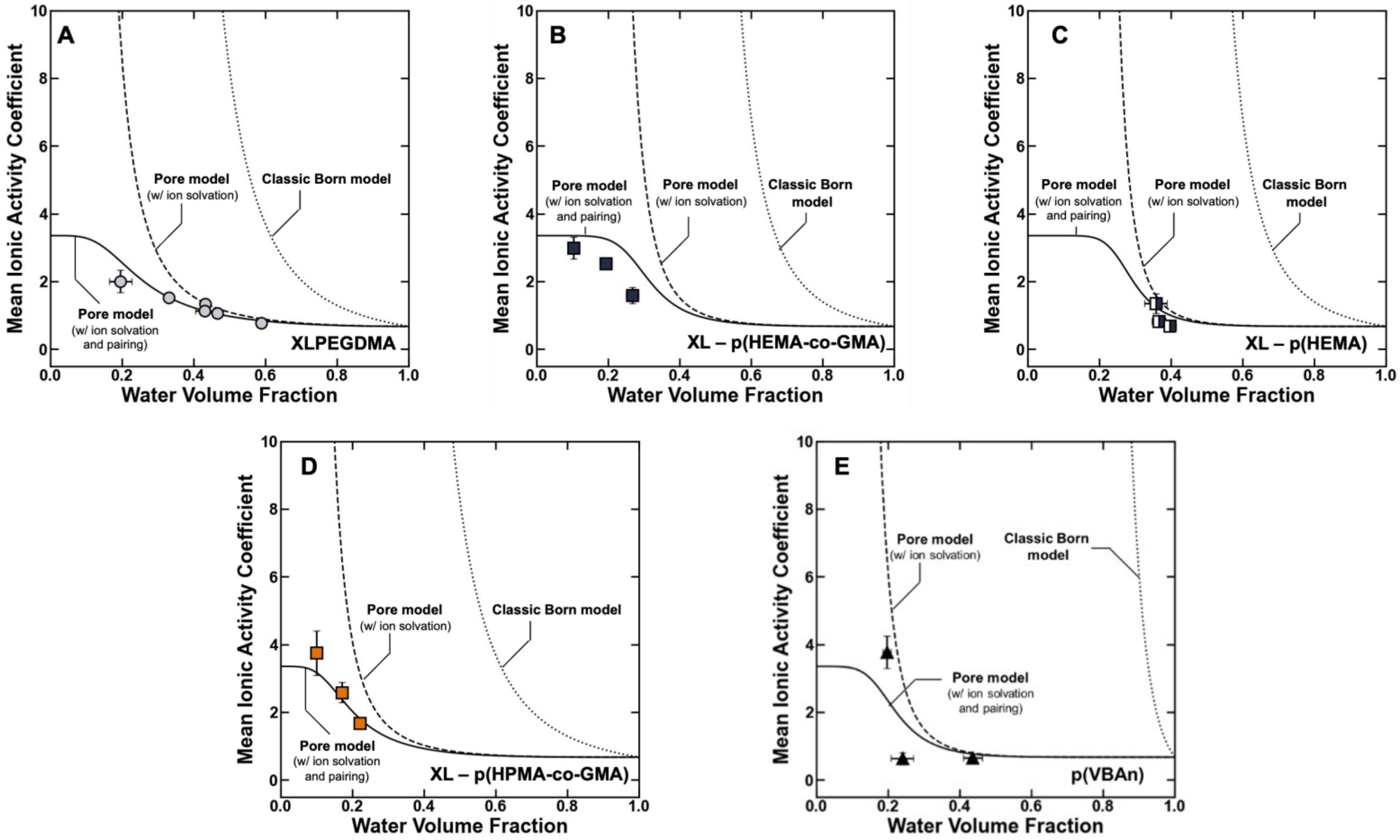

Figure S2. The experimentally-determined mean ionic activity coefficients of the polymers plotted as a function of the water volume fraction for (A) XLPEGDMA (●), (B) XL – p(HEMA-co-GMA) (■), (C) XL – p(HEMA) (◨), (D) XL – p(HPMA-co-GMA) (■), and (E) p(VBAn) (▲). The lines correspond to the applications of the pore model with ion solvation and pairing interactions (—), the pore model with ion solvation interactions (---), or the classic Born model (...).

Table S2. Summary of polymer data, and input/output for the pore model with ion solvation and pairing (Eq. 20). An association fraction of 0.2 was used for the value of α_s, as discussed in the text.

Material	Polymer Dielectric Constant, ε_m	Network Mesh Size, [Å]	Characteristic ratio, A	Mean Ionic Activity Coefficient: Experimental	Mean Ionic Activity Coefficient: Predicted
**XLPEGDMA (n=9)**	11.3 ± 0.7	5.38	7.9 ± 0.5	2.0 ± 0.05	3.11
	25 ± 5.4	7.2	2.1 ± 0.5	1.5 ± 0.03	1.40
	33 ± 4.5	8.9	1.1 ± 0.2	1.3 ± 0.06	1.01
**XLPEGDMA (n=13)**	28.3	9.2	1.4	1.1 ± 0.09	1.11
	30.5	9.4	1.2	1.1 ± 0.01	1.05
	40.4	10.5	0.6	0.8 ± 0.03	0.86
**p(VBAn)**	3.8 ± 0.5	28.7	4.9 ± 0.7	3.8 ± 0.42	2.49
	5 ± 0.6	35	3.0 ± 0.3	0.6 ± 0.16	1.77
	6.5 ± 0.8	210	0.4 ± 0.0	0.6 ± 0.02	0.78
**XL – p(HEMA-co-GMA)**	5.4 ± 0.6	6.8	14.2 ± 1.5	2.9 ± 0.25	3.34
	7.5 ± 0.9	8.5	8.0 ± 0.9	2.5 ± 0.05	3.12
	12.2 ± 0.6	11	3.5 ± 0.2	1.6 ± 0.21	1.99
**XL – p(HEMA)**	15.7 ± 0.4	18.6	1.5 ± 0.0	1.3 ± 0.29	1.17
	18.6 ± 1.1	20.1	1.1 ± 0.1	0.8 ± 0.01	1.03
	22.9 ± 3.4	26.9	0.6 ± 0.1	0.7 ± 0.10	0.86
**XL – p(HPMA-co-GMA)**	5.8 ± 0.3	6.91	13.0 ± 0.6	3.7 ± 0.57	3.33
	10.2 ± 1.1	8.2	5.8 ± 0.6	2.6 ± 0.24	2.75
	12.3 ± 1.2	9.43	4.1 ± 0.4	1.8 ± 0.09	2.20
